# Combinatorial Delivery of Docetaxel- and Erlotinib-Loaded Functionalized Nanostructured Lipid Carriers for the Treatment of Triple-Negative Breast Cancer Using Quality-by-Design Approach

**DOI:** 10.3390/pharmaceutics16070926

**Published:** 2024-07-11

**Authors:** Aiswarya Chaudhuri, Dulla Naveen Kumar, Saurabh Kumar Srivastava, Dinesh Kumar, Umesh Kumar Patil, Avanish Singh Parmar, Sanjay Singh, Ashish Kumar Agrawal

**Affiliations:** 1 Department of Pharmaceutical Engineering and Technology, IIT (BHU), Varanasi 221005, India; aiswaryachaudhuri.rs.phe20@itbhu.ac.in (A.C.); dullanaveenkr.rs.phe20@itbhu.ac.in (D.N.K.); dinesh.phe@iitbhu.ac.in (D.K.); ssingh.phe@iitbhu.ac.in (S.S.); 2 Department of Physics, IIT (BHU), Varanasi 221005, India; saurabhkrsrivastava.rs.phy19@itbhu.ac.in (S.K.S.); asparmar.phy@iitbhu.ac.in (A.S.P.); 3 Department of Pharmaceutical Sciences, Dr. Harisingh Gour Vishwavidyalaya, Sagar 470003, India; umeshpatil29@gmail.com; 4 Dr. Shakuntala Misra National Rehabilitation University, Lucknow 226017, India

**Keywords:** docetaxel, erlotinib, nanostructured lipid carriers, triple-negative breast cancer, quality-by-design (QbD), synergistic, folic acid, targeted

## Abstract

This study explored the combined administration of docetaxel (DOC) and erlotinib (ERL) using nanostructured lipid carriers (NLCs), with folic acid (FA) conjugation to enhance their synergistic anticancer efficacy against triple-negative breast cancer. NLCs were developed through hot melt homogenization–ultrasound dispersion, and optimized by a quality-by-design (QbD) approach using Plackett–Burman design and Box–Behnken design. Plots were generated based on maximum desirability. Spherical, nanosized dispersions (<200 nm) with zeta potential ranging from −16.4 to −14.15 mV were observed. These nanoformulations demonstrated ~95% entrapment efficiency with around 5% drug loading. Stability tests revealed that the NLCs remained stable for 6 months under storage conditions at 4 °C. In vitro release studies indicated sustained release over 24 h, following Higuchi and Korsmeyer–Peppas models for NLCs and FA NLCs, respectively. Additionally, an in vitro pH-stat lipolysis model exhibited a nearly fivefold increase in bioaccessibility compared to drug-loaded suspensions. The DOC–ERL-loaded formulations exhibited dose- and time-dependent cytotoxicity, revealing synergism at a 1:3 molar ratio in MDA-MB-231 and 4T1 cells, with combination indices of 0.35 and 0.37, respectively. Co-treatment with DOC–ERL-loaded FA NLCs demonstrated synergistic anticancer effects in various in vitro assays.

## 1. Introduction

Breast cancer stands as a leading cause of mortality in women, with approximately 297,790 newly diagnosed cases and an estimated 43,170 deaths in 2023, ranking it second among global cancer-related fatalities [1]. Among breast cancer types, triple-negative breast cancer (TNBC) represents the rarest and most aggressive subtype, comprising 10–15% of total cases. TNBC lacks standard hormonal receptors, rendering endocrine treatments ineffective and leaving chemotherapy as the primary therapeutic option [2]. In this context, taxanes, specifically paclitaxel and docetaxel, serve as widely employed chemotherapeutic agents against TNBC. Docetaxel (DOC), a second-generation taxane, exhibits heightened potency compared to paclitaxel, preventing microtubule depolymerization and disrupting the cell cycle at the G2 and M phases, ultimately inducing cell death [3]. Multi-omics approaches have unveiled various molecular pathways implicated in TNBC progression, presenting potential targets for therapy [4]. Epidermal growth factor receptor (EGFR) overexpression is observed in 60% of TNBC cases [5]. EGFR’s autophosphorylation activates downstream pathways, such as PI3K/AKT and RAS/MAPK, associated with cell survival, proliferation, and drug resistance in TNBC. EGFR tyrosine kinase inhibitors (EGFR TKIs) and monoclonal antibodies (mAbs) are used in the treatment of cancers with activated EGFR mutations [6]. Combination therapy gains prominence over monotherapy in cancer treatment due to its potential to enhance therapeutic efficiency by targeting multiple pathways simultaneously, providing a synergistic mechanism while limiting dose-related toxicity [7]. Numerous studies have explored combination therapies for TNBC, demonstrating synergistic effects. For instance, the combination of docetaxel and cisplatin, as well as docetaxel and carboplatin, showed promising anticancer activity [8,9]. Additionally, co-delivering erlotinib with doxorubicin enhanced doxorubicin’s apoptotic activity [7]. The current study proposes a combination of docetaxel (chemotherapeutics) and erlotinib (EGFR TKIs) to potentially induce synergistic anticancer activity against TNBC. However, differences in physicochemical and pharmacokinetic attributes between the drugs have limited their application in combination therapy. To address this, various nanoparticles were fabricated to homogenize the delivery of multiple therapeutic agents at appropriate doses, aiming for an enhanced synergistic effect with reduced toxicity in healthy organs and tissues [10]. Nanostructured lipid carriers (NLCs), second-generation lipid-based nanoparticles, have evolved to overcome drawbacks associated with first-generation lipid nanoparticles like solid lipid nanoparticles (SLNs). NLCs comprise a lipid matrix, a blend of solid and liquid lipids, stabilized by surfactants. The inclusion of a liquid lipid within the matrix results in an imperfect, less organized structure, facilitating increased loading capacity and preventing drug leakage during storage [3,11].

Moreover, it has been noted that triple-negative breast cancer (TNBC) exhibits elevated folate receptor expression in comparison to normal cells. Therefore, the conjugation of nanostructured lipid carriers (NLCs) with folic acid (FA) enhances tumor site specificity [12]. In this study, our objective is to formulate a combination of docetaxel (DOC) and erlotinib (ERL) encapsulated in NLCs and functionalized with FA to achieve a synergistic anticancer effect with enhanced targeting precision. The material characteristics and process variables essential for fabricating NLCs for oral delivery were systematically screened using the Plackett–Burman design. Subsequently, optimization was conducted using the Box–Behnken design (BBD), and the formulations were assessed for physicochemical parameters, long-term stability, in vitro drug release under simulated gastrointestinal conditions, and relevant attributes in in vitro cell culture settings.

## 2. Materials

Docetaxel was generously provided by Fresenius Kabi Oncology Ltd. (Gurgaon, India), while erlotinib was acquired from BLD Pharmatech (India) Pvt. Ltd. (Hyderabad, India). The lipids were received as gift samples from Gattefossé India Pvt. Ltd. (Mumbai, India). Tween and analytical grade chemicals were procured from Merck, Mumbai, India, while Poloxamer 407 was obtained from BASF Ltd. (Navi Mumbai, India).

## 3. Cell Lines

MDA-MB-231 cell lines were obtained from the NCCS, Pune, India, and 4T1 cell lines were graciously provided by Dr. Virander S. Chauhan, Arturo Falaschi Emeritus Scientist, ICGEB, New Delhi, India.

## 4. Methods

### 4.1. Screening of Excipients

a. Screening of solid lipid and liquid lipid: The screening of solid and liquid lipid was based on the solubility profile of the drugs in the respective lipids. An excess quantity of drugs (~10–15 mg) was added to 500 mg of each solid lipid and stirred for 1 h at a temperature 10 °C above the melting point of that solid lipid. Upon complete solubilization, ~1–2 mg of excess drugs was gradually added to the drug–solid lipid mixture until equilibrium solubility was achieved. Drug solubility in the melt lipid was visually estimated by establishing the achievement of a clear solution and the absence of drug crystals. Similarly, ~10–15 mg of drugs was added to 2 mL of liquid lipids, followed by gentle agitation in a mechanical shaker for 48 h at ambient temperature. Following this, centrifugation was performed for 20 min at 5000 rpm. Subsequently, the supernatant was extracted and diluted with methanol. Quantification of DOC and ERL in each liquid lipid was accomplished using a UV-vis spectrophotometer (Cary 60 UV-Vis spectrophotometer, Agilent, Santa Clara, CA, USA) at 230 nm and 335 nm, respectively [13].

b. Miscibility of solid and liquid lipid systems to form a binary mixture (BM): To assess the miscibility between the solid and liquid lipid, various weight-to-weight (*w*/*w*) ratios (9:1, 8:2, 7:3, 6:4, and 5:5) were employed. The mixture was heated to a temperature 10 °C above the melting point of the solid lipid. After 1 and 24 h of solidification, the blend was examined for any indication of phase separation. Additionally, the solid-state characteristics and melting point of the selected BM were analyzed using a differential scanning calorimeter (DSC-60 Plus, M/s Shimadzu (Asia Pacific) Ptv. Ltd., Tokyo, Japan) [14].

c. Screening of surfactant: Surfactants were screened based on the emulsification ability of the surfactant for the BM. BM (100 mg) was dissolved in 3 mL methylene chloride, which was then added to the 5% *w*/*v* surfactant solutions (10 mL), followed by stirring at 40 °C in a magnetic stirrer. Then, 1 mL of the mixture was diluted with distilled water and percentage transmittance was determined using the UV-vis spectrophotometer ((Cary 60 UV-Vis spectrophotometer, Agilent, CA, USA) at 638.2 nm [15].

### 4.2. Compatibility Study

Compatibility between drugs and the excipients was analyzed by Fourier-transform infrared (FTIR) spectroscopy (Shimadzu FTIR 8300 Spectrophotometer, Tokyo, Japan) [16].

### 4.3. Preparation of DOC–ERL NLCs

DOC–ERL NLCs were prepared by the hot melt homogenization–ultrasound dispersion method as previously reported with slight modification [3]. BM was melted at 70 °C, and accurately weighed drugs (0.1% *w*/*v* for DOC, and 0.3% *w*/*v* for ERL) were added to the melted lipid. Simultaneously, the aqueous phase was prepared by dissolving Tween 20 in distilled water at 70 °C. The aqueous phase was added dropwise and stirred in a magnetic stirrer for 1 h at 750 rpm and 70 °C. The resultant coarse emulsion was then subjected to high-speed homogenization (IKA T-25 digital ultra Turrax, Staufen, Germany) for 1 min at 5000 rpm, followed by probe sonication (UP 200H, Hielscher, Teltow, Germany) for a specific time at 60% amplitude and cooled at room temperature for lipid solidification and formation of NLCs. The NLCs prepared were then stored at 4 °C for characterization.

### 4.4. Optimization of DOC–ERL NLCs

A QbD approach was employed to assess the effects of critical attributes associated with formulation and instrumental variables. Figure S1 shows the Ishikawa diagram used as a visualization tool for the QbD approach.

A seven-factor, eighteen-run PBD was applied for screening the various formulations and process variables of DOC–ERL NLCs, and three-factor, three-level BBD was employed to optimize the varied response variables using SAS JMP^®^ Pro 14 (trial version). The details of experimental batches of NLCs with PBD are given in Table S1. The factors and responses for PBD and BBD are given in Table 1.

### 4.5. Preparation of FA–DOC–ERL NLCs

Folate-conjugated DOC–ERL NLCs (FA–DOC–ERL NLCs) were prepared using the same method described in Section 4.3, except by adding 6 mg of an FA–SA conjugate in the lipid phase. The FA–SA conjugate was synthesized through an EDC coupling reaction following a previous report with slight modifications [17]. First, 200 mg stearic acid and 200 mg EDC were added to 5 mL DMF and stirred thoroughly for 1 h at ambient temperature. Then, 300 mg folic acid solution in 1.5 mL pyridine was added to this mixture and the reaction was stirred for 18 h at ambient temperature. To precipitate the conjugate, distilled water was added to the reaction mixture. The water-soluble by-products, EDC, and any unreacted folic acid were removed by dialyzing the mixture using a membrane with a molecular weight cutoff of 12–14 kDa (Sigma Aldrich, Bangalore, India) for 72 h in distilled water. The water was changed every 6 h with fresh distilled water. The resulting solution was then filtered through a 0.45 μm Millipore filter and subjected to lyophilization to obtain the yellow FA–SA conjugate. Figure S2A shows a schematic diagram of the preparation of the FA–SA conjugate. Formation of the FA–SA conjugate was confirmed using FTIR (Nicolet iS5, Thermo Electron Scientific Instruments LLC, Tokyo, Japan), as shown in Figure S2B.

### 4.6. Characterization of NLCs

#### 4.6.1. Particle Size (PS), Polydispersity Index (PDI), and Zeta Potential (ZP)

PS, PDI, and ZP were measured using dynamic light scattering (DLS) using a Zetasizer (DelsaTMNano, Beckman Coulter, Brea, CA, USA). Briefly, prior to analysis, small aliquots of different formulations were diluted with distilled water and measured for particle size, PDI, and ZP with the Zetasizer [18].

#### 4.6.2. Entrapment Efficiency and Drug Loading

The percentage of drugs entrapped and the drug loading efficiency were determined indirectly by measuring the concentration of free drugs in the supernatant [18,19]. A 2 mL formulation was ultracentrifuged at 45,000 rpm for 30 min at 4 °C, and the supernatant was collected and the concentrations of drugs were analyzed by the validated HPLC method with an Agilent 1260 Infinity II HPLC system (Santa Clara, CA, USA). EE and DL were calculated using Equations (1) and (2).

(1) $$\%\;Entrapment\;efficiency=\frac{Total\;amount\;of\;drug-amount\;of\;drug\;in\;the\;supernatant}{Total\;amount\;of\;drug\;}\;\times \;100$$

(2) $$\%\;Drug\;loading=\frac{Total\;amount\;of\;drug-amount\;of\;drug\;in\;the\;supernatant}{Total\;weight\;of\;the\;nanoparticles\;}\;\times \;100$$

#### 4.6.3. Morphology Analysis

The shape and morphology of the different formulations were determined using transmission electron microscopy (TEM Tecnai G2 20 TWIN, FEI Company of USA (SEA) Pte, Ltd., Los Angeles, CA, USA) [20] and atomic force microscopy (AFM NTEGRA Prima, NT-MDT Service and Logistics Ltd., Amsterdam, The Netherlands) [18]. For TEM analysis, the NLCs were first suitably diluted with distilled water, after which a drop was placed on the carbon-coated grid, air-dried, and analyzed using TEM. For AFM analysis, the NLCs were suitably diluted with distilled water, followed by the addition of dispersions (10 μL) onto a clean glass slide, air-dried, and analyzed under AFM.

#### 4.6.4. Fourier Transform Infrared Spectroscopy–Attenuated Total Reflectance (FTIR-ATR)

The differences within the functional moieties of the formulations were observed using Fourier-transform infrared spectroscopy (Nicolet iS5, Thermo Electron Scientific Instruments LLC, Japan) [21].

### 4.7. Freeze-Drying Studies

Different NLC formulations were freeze-dried, as per our previous report [20]. A preliminary screening of the cryoprotectants was performed where the NLC dispersions were mixed with 5% *w*/*v* of different cryoprotectants. After screening the cryoprotectant, the one selected was subjected to different concentrations (2.5–10% *w*/*v*). The freeze-dried NLC formulations were analyzed in terms of the re-dispersibility index and re-dispersibility score.

### 4.8. In Vitro Drug Release

An in vitro release study was performed in simulated gastric fluid (pH 1.2) for 2 h, intestinal fluid (pH 6.8) for 6 h, and PBS (pH 7.4, and pH 5.5) for 24 h by the dialysis membrane method [17]. The study was performed in simulated gastric fluid (pH 1.2) for 2 h, intestinal fluid (pH 6.8) for 6 h, and PBS (pH 7.4, and pH 5.5) for 24 h by the dialysis membrane method. The sink condition was maintained by 0.1% *w*/*v* Tween 80. The NLCs (equivalent to 2 mg of drugs) were added to an activated dialysis bag (MWCO: 12 kDa), and suspended in 50 mL of each release medium at 37 ± 0.5 °C and 100 rpm. Aliquots of 1 mL were withdrawn at different time intervals and replaced with an equal volume of fresh media. The withdrawn samples were then analyzed using the validated HPLC method (Agilent 1260 Infinity II, Santa Clara, CA, USA). To evaluate the drug release kinetics, the in vitro drug release data were fitted to five mathematical kinetic models: zero order, first order, Higuchi model, Hixson–Crowell model, and Korsmeyer–Peppas model [22].

### 4.9. In Vitro Lipolysis Study

In vitro lipolysis was performed according to previous reports with slight modifications [23,24]. The NLC dispersion was mixed with 9 mL lipolysis buffer and allowed to stir at 200 rpm at 37 ± 1 °C for 10 min while its pH was maintained at 6.8 ± 0.2 using 1 M NaOH or HCl. Then, 1 mL of chilled pancreatin was added to initiate the enzymatic digestion of the formulation, and the pH of the medium was maintained at 6.8 ± 0.2 pH using 0.2 M NaOH solution. The stated temperature and the pH were maintained throughout the study to neutralize the liberated free fatty acids produced upon lipid digestion, as evidenced by the decreased pH. The experiment was continued for 2 h, and a volume of 0.2 M NaOH added to neutralize the free fatty acids, which were calculated using Formula (3):

(3) $$\%FFA=\;\frac{V_{NaoH}\;\times \;m_{NaOH}\;\times \;{\mathrm{Mw}}_{lipid}\;\times \;100}{W_{lipid}\;\times \;2}$$

where V_NaOH_ = volume of NaOH in liters, m_NaOH_ = molarity of NaOH, Mw_lipid_ = apparent molecular weight of the lipid, and W_lipid_ = weight of lipid in the media in grams.

After completion of the in vitro lipolysis study, the final digestion media were treated with 1 M 4-bromophenylboronic acid in methanol (5 mL per 1 mL of digestion media) to inhibit further digestion, followed by ultracentrifugation (Type 90 Ti rotor, Beckman Coulter, Mumbai, India) at 40,000 rpm for 40 min at 4 °C, after which the digest was separated into a bottom sediment phase, a middle aqueous phase containing the formulated micelles, and a top oil phase. The middle aqueous phase was collected, mixed with an equal volume of methanol, and centrifuged for 15 min at 4000 rpm. The top methanol phase was collected, in which the drugs were solubilized. The bottom aqueous layer was again mixed with additional methanol and centrifuged, as mentioned above. The top methanol layer containing the solubilized drugs was mixed with the previously collected methanol layer, which was then analyzed using HPLC (Agilent 1260 Infinity II, Santa Clara, CA, USA). The bioaccessibility (%) of DOC and ERL was calculated using Formula (4):

(4) $$Bioaccessibility\;\left(BA\right)\%=\;\frac{Total\;mass\;of\;solubilized\;drug\;\left(g\right)\;\times \;100}{Total\;mass\;of\;drug\;in\;original\;lipid\;sample\;\left(g\right)}$$

### 4.10. Stability Study

Freeze-dried NLCs were evaluated for storage stability as per ICH guideline Q1A (R2), where the lyophilized NLCs were transferred to glass vials and stored at 25 ± 2 °C/60 ± 5%RH and 40 ± 2 °C/75 ± 5%RH, and 4 °C. The stability study was conducted for six months. The samples were withdrawn after 1, 3, and 6 months and compared with the freshly prepared NLCs [25].

A stability study of NLCs with different pH was also performed. Briefly, the NLCs were subjected to different pH to assess their stability in pH changes for 1 week at 4 °C, followed by measurement of particle size. Each sample was tested in triplicate [26].

### 4.11. Cell Viability Assay

The cell viability was analyzed in MDA-MB-231 and 4T1 cell lines using a colorimetric-based MTT assay. Briefly, 1 × 10^4^ cells/well were seeded in a 96 well plate and incubated overnight, followed by treatment with different doses of DOC, ERL, and different NLC dispersions for 24, 48, and 72 h. MTT treatment (0.5 mg/mL) was applied after these periods and cells incubated for 4 h, followed by DMSO to dissolve the formazan crystals [21]. The percentage cell viability was calculated using Equation (5):

(5) $$\%\;Viability=\;\frac{\left(Abs\;of\;Sample\;well\right)\times 100}{Abs\;of\;control\;well}$$

wherein Abs of the control is the absorbance of the control group and Abs of the sample is the absorbance of the sample.

#### Determination of Combination Effect

Based on the IC_50_ value of the DOC and ERL, the drugs were mixed in different molar ratios, viz. 1:4, 1:3, 1:2, 1:1, 2:1, 3:1, and 4:1. The IC_50_ of the combination was obtained, which was further employed for identification of combination indices (CIs). The CompuSyn program (ComboSyn, Inc., Paramus, NJ, USA) analyzed the data [27,28].

### 4.12. Quantitative and Qualitative Cellular Uptake Assay

A quantitative cell uptake assay was performed as per previous reports [18,29]. The amount of drugs taken up by the cells was quantified using HPLC (Agilent 1260 Infinity II HPLC system). The cells (5 × 10^5^ cells/well) were seeded and incubated overnight, followed by treatment with DOC, ERL, and different NLC dispersions (equiv. 2 μM DOC and 20 μM ERL), then incubated for 0.5, 1, 2, and 4 h. The cells were washed with chilled PBS, followed by trypsinization, and centrifugation at 1600 rpm for 5 min. The pellets were treated with methanol, followed by centrifugation at 10,000 rpm for 10 min. The supernatant obtained was then analyzed using HPLC (Agilent 1260 Infinity II HPLC system).

For qualitative analysis, coumarin 6 (C6) was used as a model dye to evaluate the qualitative cellular uptake of drugs in MDA-MB-231 cells. Briefly, 3 × 10^5^ cells/well were seeded and incubated for 24 h, followed by treatment with C6, C6-loaded NLCs, and C6-loaded FA NLCs (C6 equiv. 3 μg), and incubated for 4 h. The cells were washed with chilled PBS, treated with DAPI (10 μg/mL), and again incubated for 10 min. The cells were then observed under a fluorescence microscope (EVOS FLoid) [30].

### 4.13. Mitochondrial Membrane Potential (MMP) Assay

The mitochondrial membrane potential (MMP) was analyzed using JC-1 dye (Invitrogen, Thermo Scientific, San Diego, MA, USA) [31]. Briefly, cells (1 × 10^5^ cells/well) were seeded in a 12-well plate and incubated overnight, followed by treatment with drugs and different NLC dispersions (equiv. to 2 μM DOC and 20 µM ERL when administered alone and 2 μM DOC and 6 µM ERL in combination) and incubated for 24 h. The cells were washed with chilled PBS and incubated with JC-1 dye (10 μM) for 30 min, followed by washing with chilled PBS and analysis under a fluorescence microscope (Olympus BX53, Tokyo, Japan) [32].

### 4.14. Colony Formation Assay

MDA-MB-231 cells were seeded at 500 cells/well and incubated overnight, followed by treatment with drugs and different NLC dispersions (equiv. 0.5 μM DOC and 8 µM ERL when administered alone and equiv. 0.5 μM DOC, and 1.5 µM ERL in combination) and incubated. The study was continued for 10 days. After every 24 h, the media were replaced with fresh media. After completion of the study, the cell colonies were stained with 0.5% crystal violet in methanol for 10 min. The number of colonies was manually counted under a microscope and recorded by a digital camera [33].

### 4.15. Transwell Migration Assay

To assess the anti-migration capacity of the formulations, a transwell cell migration assay was performed using 6 transwell chambers (Sigma Aldrich, Burlington, MA, USA). Briefly, cells were pretreated with the different formulations (equiv. 2 μM DOC and 20 µM ERL when administered alone and 2 μM DOC and 6 µM ERL in combination) for 1 h, followed by trypsinization. Then, 4 × 10^2^ cells in 100 µL non-serum media were seeded in the upper chambers of the transwell. The lower chamber was filled with complete media, followed by incubation for 24 h. The migrated cells were fixed using 4% formaldehyde and stained with 0.5% toluidine blue. The number of migrated cells was then counted using an inverted microscope (Dewinter, New Delhi, India) [34].

### 4.16. Reactive Oxygen Species (ROS) Assay

H_2_DCFDA dye was employed to analyze the generation of intracellular reactive oxygen species. Briefly, the cells were seeded and incubated overnight, followed by different treatments (equiv. to 2 μM DOC and 20 µM ERL when administered alone and 2 μM DOC and 6 µM ERL in combination), and incubated for 24 h, followed by addition of H_2_DCFDA (10 µM) and incubation for 30 min. Cells were washed with chilled PBS and observed under the fluorescence microscope (Olympus BX53, Tokyo, Japan). H_2_O_2_ (0.05%) was considered a positive control and added 2 h before the completion of the study period [35].

## 5. Statistical Analysis

All the data are expressed as means ± standard deviation (SD). The statistical analysis was performed by applying a one-way ANOVA followed by the Tukey–Kramer multiple-comparison test in Graph Pad Prism version 5.0 (GraphPad Software, Inc., San Diego, CA, USA), where *p* < 0.05 was considered statistically significant. ImageJ software Version 1.8.0 (National Institute of Health, Bethesda, MD, USA) was used to analyze the in vitro images and CompuSyn software Version 1.0 was used for the combination studies.

## 6. Results

### 6.1. Screening of Excipients

**Figure 1:**
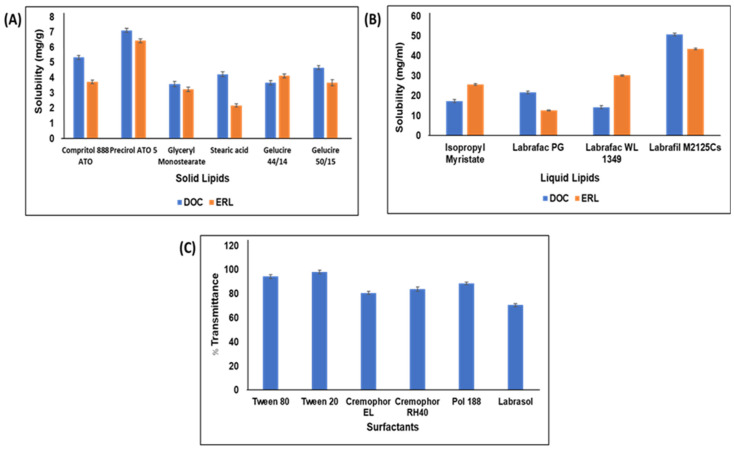
Solubility of DOC and ERL in (**A**) solid lipids and (**B**) liquid lipids. (**C**) Transmittance of BM in different surfactants.

DOC and ERL exhibited maximum solubility in Precirol ATO 5 (Figure 1A) and Labrafil M2125 Cs (Figure 1B), and hence were selected as solid and liquid lipids, respectively. The BM at a 7:3 ratio did not show any sign of turbidity or phase separation. DSC (Figure S3) showed that the 7:3 ratio lowered the solid lipid’s melting point significantly, indicating incorporation of liquid lipid into lipid matrices. Transmittance of BM dispersion was found to be highest with Tween 20 (Figure 1C).

### 6.2. Compatibility Study

Precirol ATO 5, Labrafil M2125Cs, and Tween 20 showed characteristic peaks. The physical mixtures of drugs and excipients exhibited characteristic peaks (Figure S4A,B).

### 6.3. Optimization of DOC–ERL NLCs

#### 6.3.1. Plackett–Burman Design (PBD)

**Figure 2:**
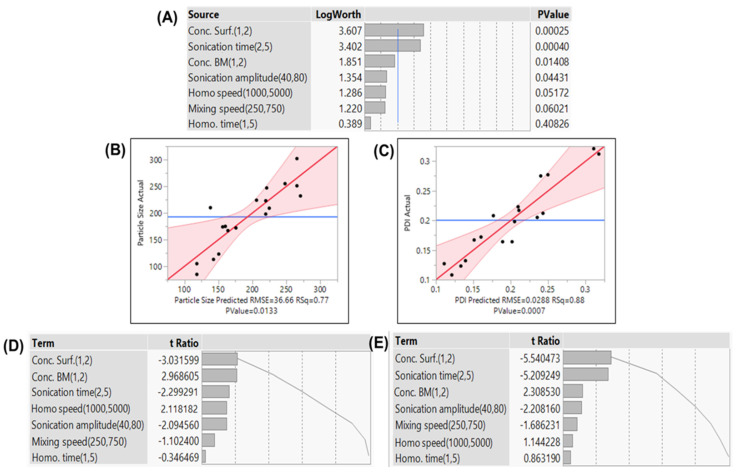
(**A**) Effect summary of the model. Actual vs. predicted plots for PS (**B**) and PDI (**C**) and Pareto plot for PS (**D**) and PDI (**E**).

In the PBD, ANOVA showed a *p*-value < 0.05 for significant factors. Surfactant concentration exhibited a significant effect on PS and PDI, while the BM concentration showed a significant effect on PS (Figure 2).

#### 6.3.2. Box–Behnken Design (BBD)

BBD suggested that the responses were significantly affected (*p*-value < 0.05) by the independent variables (factors). The details of experimental batches of DOC NLCs and ERL NLCs in BBD are given in Table 2 and Table 3, respectively.

Response surface plots were employed to analyze the interaction between the two variables and the responses while keeping the other variables constant (Figures S5A–L and S6A–L).

A comparison was performed between the experimental values and the predicted values, which then provided a 10% prediction error (Table 4).

### 6.4. Characterization of NLCs

**Figure 4:**
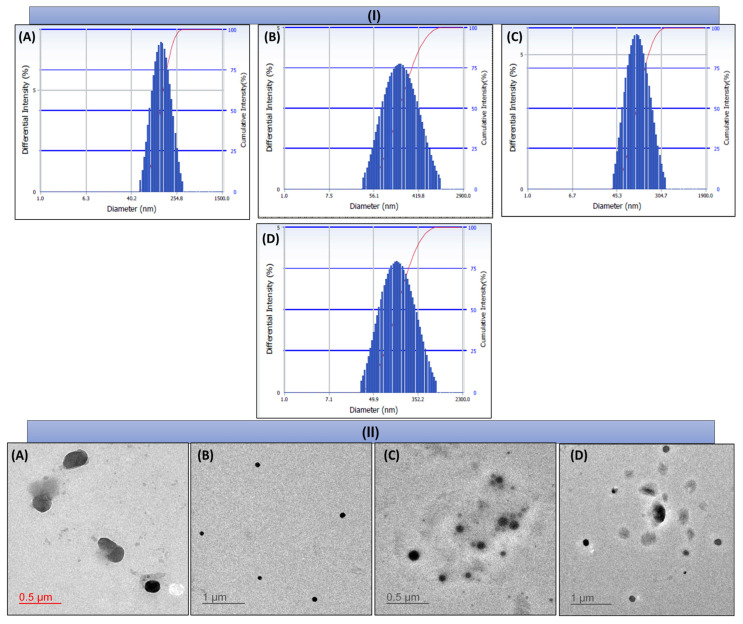

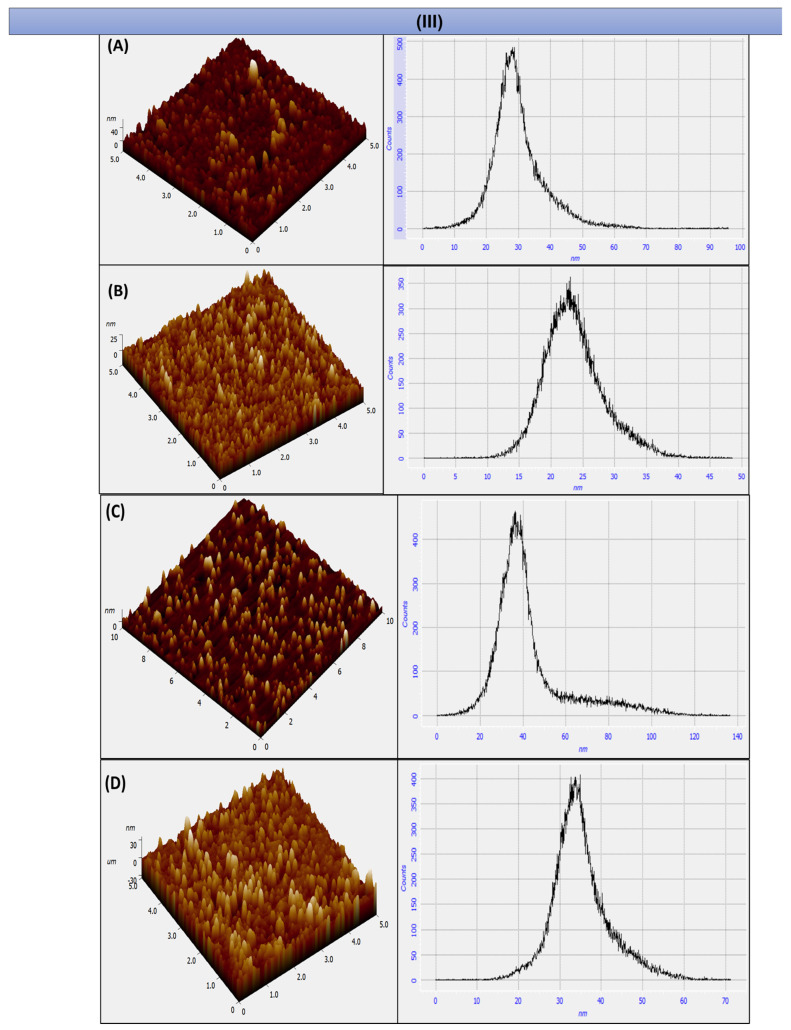
Size and morphology determination using (**I**) Zetasizer, (**II**) TEM, and (**III**) AFM of DOC NLCs (**A**), FA–DOC NLCs (**B**), ERL NLCs (**C**), and FA–ERL NLCs (**D**).

The PS, PDI, ZP, EE, and DL of different NLCs are shown in Table 5. Morphology and height were further determined by TEM (Figure 4II(A–D)) and AFM (Figure 4III(A–D)) respectively, which revealed that the particles formed were spherical and homogeneously dispersed, and they were found to be in good correlation with the analysis obtained by DLS (Figure 4I(A–D)).

### 6.5. Fourier-Transform Infrared Spectroscopy–Attenuated Total Reflectance (FTIR–ATR)

The FTIR spectra of the folic acid–stearic acid conjugate (FA–SA), different NLC dispersions, and FA-conjugated NLC dispersions were obtained. FA–SA exhibited C=O stretching vibrations (amide I band), C-N stretching, and N-H bending vibrations (2° amide II band), and NH stretching vibrations (amide A band) at 1684 cm^−1^, 1535 cm^−1^, and 3407 cm^−1^ respectively. The stated bands of amide linkage appeared in FA–DOC NLCs and FA–ERL NLCs, but were absent in DOC NLCs and ERL NLCs, which suggested the successful conjugation of folic acid onto the surface of NLCs (Figure S7).

### 6.6. Freeze-Drying

The influence of freeze-dried formulations on quality attributes is shown in Table S2. Based on the re-dispersity index, fructose was selected as the cryoprotectant. It was found that 5% *w*/*v* showed a re-dispersity index close to 1 (Table S3).

### 6.7. In Vitro Drug Release

**Figure 5:**
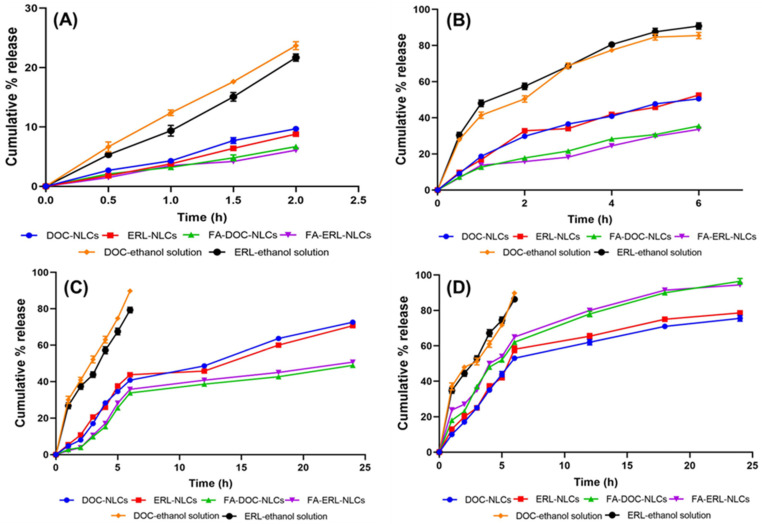
Comparative release profile of DOC NLCs, ERL NLCs, FA–DOC NLCs, FA–ERL NLCs, DOC–ethanol solution, and ERL–ethanol solution in different simulated biological media: (**A**) SGF (pH 1.2), (**B**) SIF (pH 6.8), (**C**) PBS (pH 7.4), and (**D**) PBS (pH 5.5) at 37 °C. Values are presented as means ± SD (n = 3).

Figure 5 illustrates the in vitro release profiles of various NLCs. Notably, ~90% of the drugs were released from NLCs at pH 5.5, while ~80% of the drugs were released at pH 7.4, all within a 24 h timeframe. At pH 6.8, 50% of the drugs were released within 6 h, and <20% of the drugs were released at pH 1.2 within 2 h. A slight increase in drug release was noted at pH 5.5 for FA-conjugated NLCs compared to the unconjugated NLCs.

The model fitting analysis revealed that DOC NLCs and ERL NLCs adhere to the Higuchi kinetic model, whereas FA–DOC NLCs and FA–ERL NLCs align with the Korsmeyer–Peppas model. Further examination of the Korsmeyer–Peppas model indicated that all DOC–ERL NLCs conform to a Fickian transport mechanism, while DOC–ERL–FA NLCs exhibit a non-Fickian drug transport mechanism (Table S4).

### 6.8. In Vitro Lipolysis

**Figure 6:**
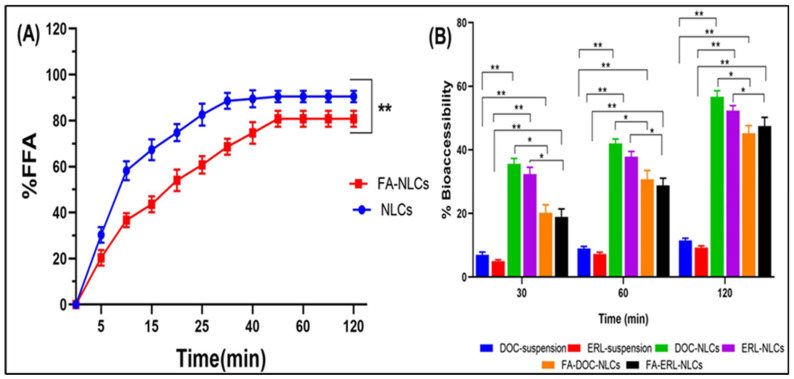
(**A**) Lipid digestion kinetics from NLCs and FA NLCs, expressed as %FFA as a function of time. (**B**) Bioaccessibility of DOC and ERL from suspension, NLCs, and FA NLCs after in vitro lipolysis. Data are presented as means ± SD (n = 3). Statistically significant ** *p* < 0.01, and * *p* < 0.05.

The cumulative lipolysis percentage was observed in terms of liberation of free fatty acids (FFAs), as shown in Figure 6A. It was observed that NLCs showed relatively fast lipolysis within the first 20 min (74.8 ± 3.7%), compared to FA NLCs (54 ± 4.7%), and then proceeded with slow digestion for both formulations. At the end of the study, the FFAs released for NLCs amounted to 90.5 ± 2.5%, and for FA NLCs, this was 80.8 ± 3.5%. It was observed that the bioaccessibility of drugs from the suspension was lowest due to their lipophilic nature. The BA of the drugs was increased when delivered through NLCs. However, the BA was slightly lowered in FA-conjugated NLCs (Figure 6B).

### 6.9. Stability Study

The impact of various temperature and humidity conditions on the critical quality attributes of NLCs is presented in Table S5. No significant changes (*p* > 0.05) were noted in the quality attributes of the formulations at 4 °C or 25 ± 2 °C/60 ± 5%RH. However, a significant change (*p* < 0.05) was observed in the quality attributes of the formulations at 40 ± 2 °C/75 ± 5%RH.

Particle size showed a few changes in different media; however, we considered that all NLCs were stable during all experiments according to the results (Figure S8).

### 6.10. Cell Viability Assays

**Figure 7:**
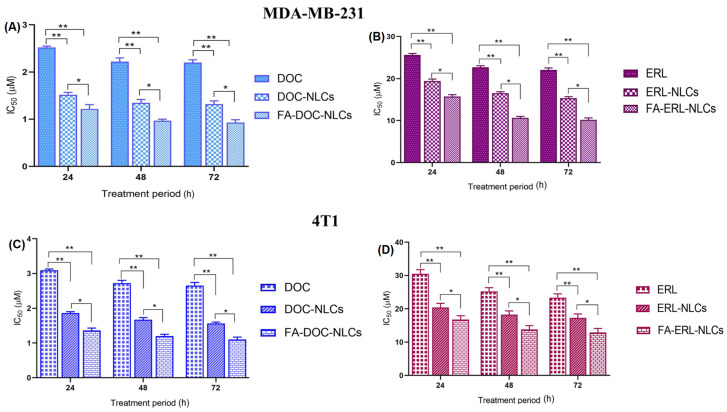
IC_50_ values of DOC-loaded NLCs in MDA-MB-231 (**A**) and 4T1 (**C**). IC_50_ values of ERL-loaded NLCs in MDA-MB-231 (**B**) and 4T1 (**D**). Statistical analysis was performed by one-way ANOVA followed by the Tukey P test with multiple comparisons and a significance level of * *p* < 0.05, and ** *p* < 0.01.

Cell viability assays were performed to evaluate the cytotoxicity of different NLC dispersions in triple-negative breast cancer cell lines. A significant reduction in IC_50_ (*p* < 0.01) of drugs loaded with FA NLCs was observed compared to free drugs (Figure 7). Cell viability at different times is shown in Figures S9 and S10 for MDA-MB-231 and 4T1, respectively.

#### Determination of Combination Effect

**Figure 8:**
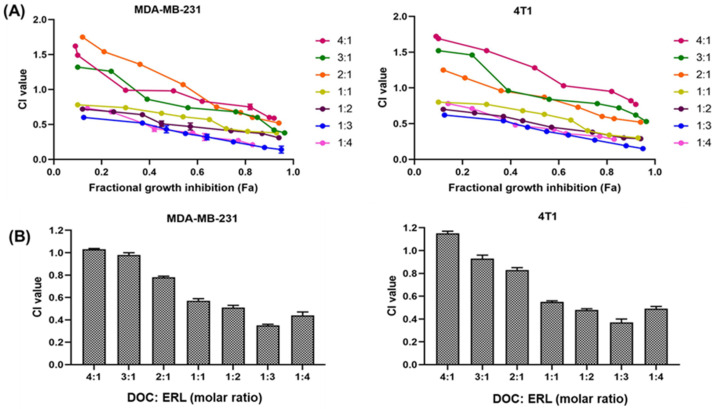
(**A**) Screening of DOC and ERL combination ratio for MDA-MB-231 and 4T1 cells. (**B**) Comparison of CI values of different ratios of DOC and ERL in MDA-MB-231 and 4T1 cells.

The combination of DOC and ERL showed synergistic activity in MDA-MB-231 and 4T1 cell lines at a molar ratio of 1:3, with a lowest CI value of 0.35 and 0.37 for MDA-MB-231 and 4T1, respectively (Figure 8). The IC_50_ of the combinations is demonstrated in Table 6.

### 6.11. Quantitative and Quantitative Cellular Uptake

**Figure 9:**
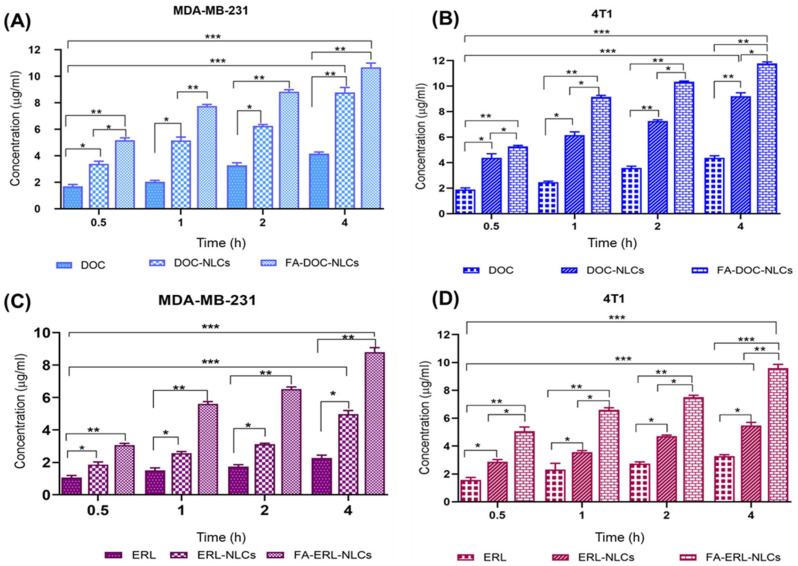
Quantitative cellular uptake assay of DOC, DOC NLCs, FA–DOC NLCs, ERL, ERL NLCs, and FA–ERL NLCs after 0.5, 1, 2, and 4 h incubation periods in MDA-MB-231 (**A**,**C**) and 4T1 cell (**B**,**D**) lines. Statistically significant at * *p* < 0.05, ** *p* < 0.01, and *** *p* < 0.001.

Significant cellular uptake of DOC and ERL was observed from drug-loaded FA NLCs compared to free drugs and non-functionalized drug-loaded NLCs (Figure 9).

**Figure 10:**
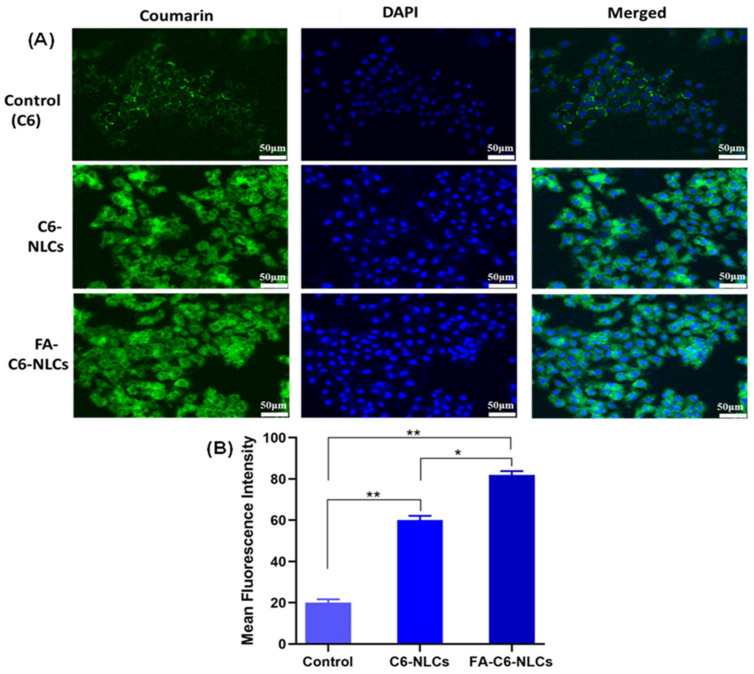
(**A**) Visual representation of the qualitative cellular uptake of coumarin 6 (C6) and various types of NLCs on MDA-MB-231 cells, observed through a fluorescence microscope. (**B**) Quantitative analysis of mean fluorescence intensity corresponding to C6 and different types of NLCs, determined using coumarin 6 dye. Statistical analysis was conducted through one-way ANOVA followed by the Tukey post hoc test for multiple comparisons, with significance levels denoted as * *p* < 0.05 and ** *p* < 0.01.

Similarly, the cellular accumulation of FA-C6 NLCs was found to be significantly higher compared to C6 NLCs and C6 (Figure 10).

### 6.12. Mitochondrial Membrane Potential (MMP) Assay

**Figure 11:**
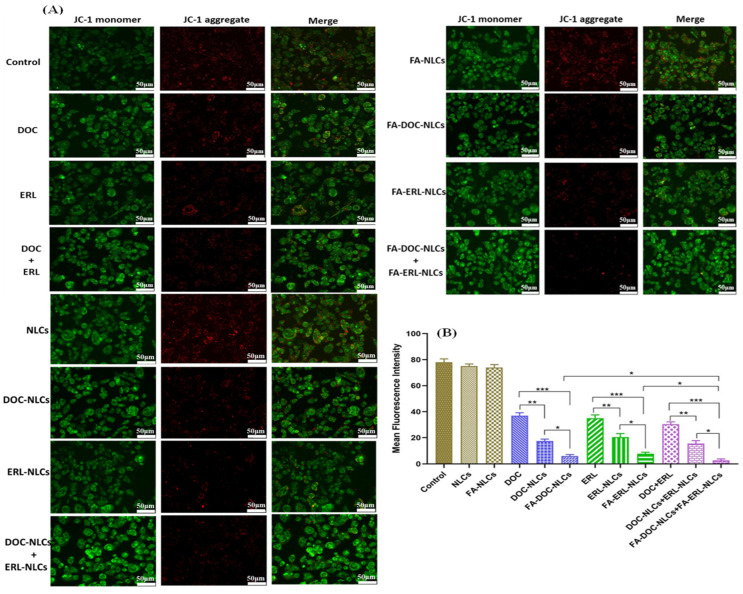
(**A**) Assessment of mitochondrial membrane potential in MDA-MB-231 cells following treatment with drugs, their combinations, various NLC dispersions, and their combinations, visualized through fluorescence. (**B**) Quantification of mean fluorescence intensity corresponding to drugs, their combinations, different NLC dispersions, and their combinations with reference to JC-1 aggregates (red fluorescence). Statistical analysis using one-way ANOVA followed by the Tukey post hoc test for multiple comparisons, with significance levels denoted as * *p* < 0.05, ** *p* < 0.01, and *** *p* < 0.001.

**Figure 12:**
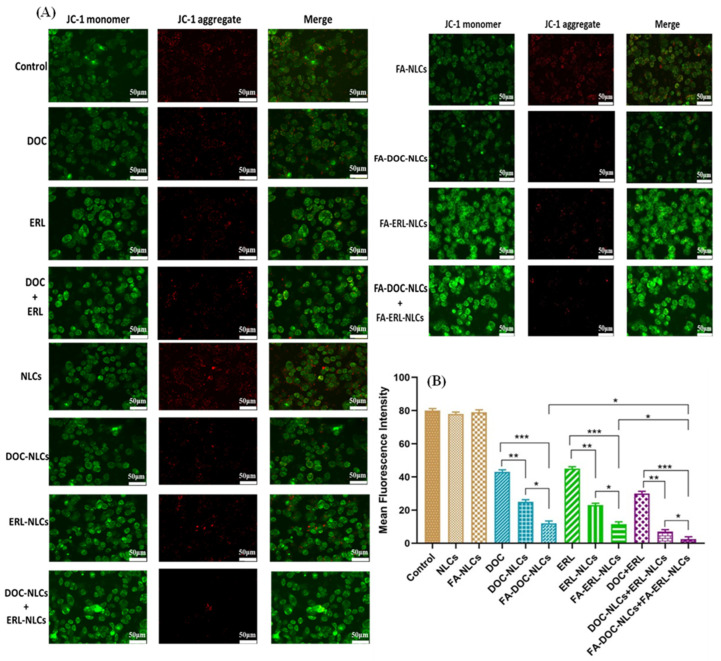
(**A**) Evaluation of mitochondrial membrane potential in 4T1 cells following treatment with drugs, their combinations, various NLC dispersions, and their combinations. (**B**) Quantification of mean fluorescence intensity corresponding to drugs, their combinations, different NLC dispersions, and their combinations with respect to JC-1 aggregates (red fluorescence). Statistical analysis was conducted using one-way ANOVA followed by the Tukey post hoc test for multiple comparisons, with significance levels denoted as * *p* < 0.05, ** *p* < 0.01, and *** *p* < 0.001.

A notable reduction in JC-1 aggregates (red fluorescence) was evident in FA–DOC NLCs + FA–ERL NLCs, in contrast to DOC + ERL and individual drugs (Figure 11 and Figure 12).

### 6.13. Colony Formation Assay

**Figure 13:**
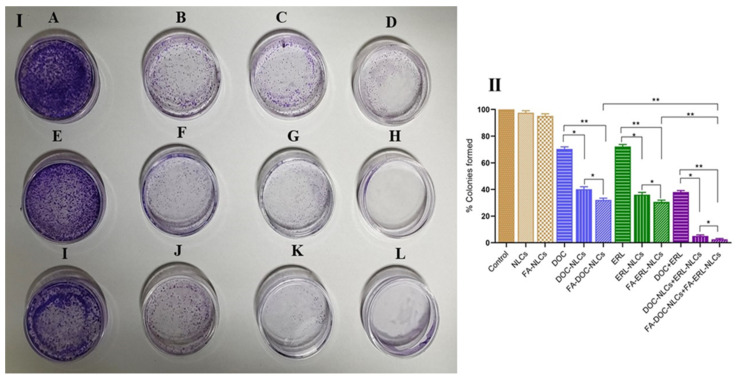
(**I**) Visual representation of the MDA-MB-231 cell colony assay with stained colonies resulting from different treatments: (**A**) control, (**B**) DOC, (**C**) ERL, (**D**) DOC + ERL, (**E**) NLCs, (**F**) DOC NLCs, (**G**) ERL NLCs, (**H**) DOC NLCs + ERL NLCs, (**I**) FA NLCs, (**J**) FA–DOC NLCs, (**K**) FA–ERL NLCs, (**L**) FA–DOC NLCs + FA–ERL NLCs. (**II**) Statistics of colony-formation assay. Statistical analysis was performed using one-way ANOVA followed by the Tukey P test with multiple comparisons and the level of significance * *p* < 0.05 and ** *p* < 0.01.

Drug-loaded NLCs exhibited a reduction in colony formation compared to single drugs (*p* < 0.05). Furthermore, the co-administration of FA-conjugated drug-loaded NLCs significantly decreased the formation of colonies compared to single administration of FA-conjugated drug-loaded NLCs (*p* < 0.01) and unconjugated drug-loaded NLCs (*p* < 0.05) (Figure 13).

### 6.14. Transwell Migration Assay

**Figure 14:**
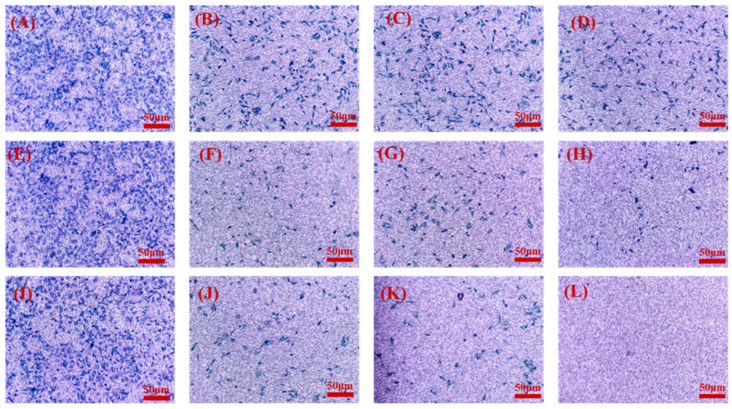
Transwell migration assay indicating the anti-migration property of various treatments over MDA-MB-231 cells: (**A**) control, (**B**) DOC, (**C**) ERL, (**D**) DOC + ERL, (**E**) NLCs, (**F**) DOC NLCs, (**G**) ERL NLCs, (**H**) DOC NLCs + ERL NLCs, (**I**) FA NLCs, (**J**) FA–DOC NLCs, (**K**) FA–ERL NLCs, (**L**) FA–DOC NLCs + FA–ERL NLCs.

The transwell migration assay was conducted to assess the anti-migratory properties of NLCs compared to single drugs. As depicted in Figure 14, co-administration of FA-conjugated drug-loaded NLCs exhibited decreased migration compared to the control, unconjugated NLCs, and single administration of conjugated NLCs.

### 6.15. Reactive Oxygen Species (ROS) Assay

**Figure 15:**
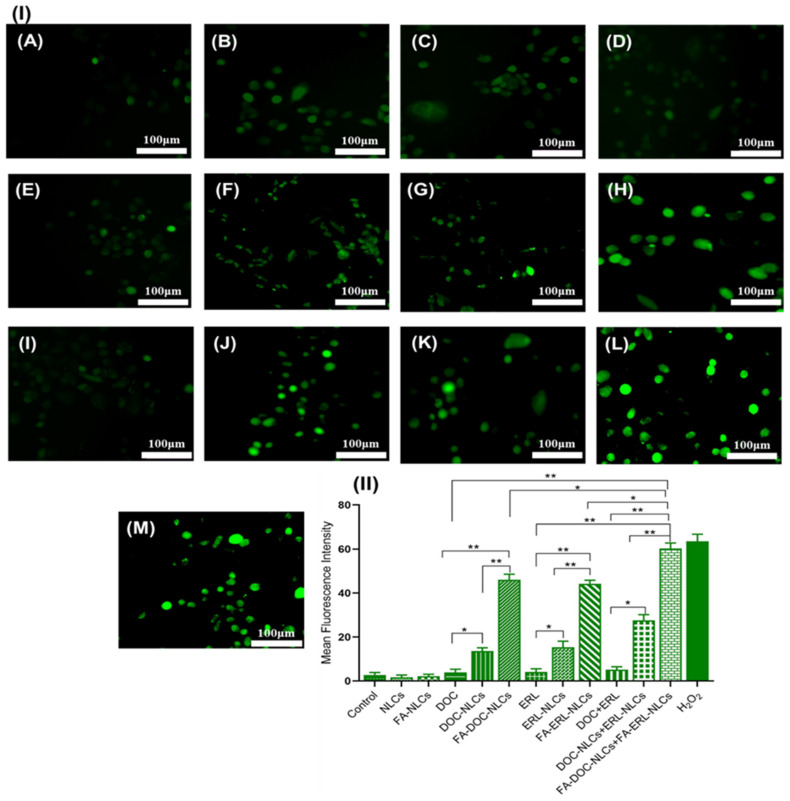
(**I**) Reactive oxygen species assay was analyzed by fluorescence microscopy using H_2_DCFDA dye (10 μM) for different treatments: (**A**) control, (**B**) DOC, (**C**) ERL, (**D**) DOC + ERL, (**E**) NLCs, (**F**) DOC NLCs, (**G**) ERL NLCs, (**H**) DOC NLCs + ERL NLCs, (**I**) FA NLCs, (**J**) FA–DOC NLCs, (**K**) FA–ERL NLCs, (**L**) FA–DOC NLCs + FA–ERL NLCs, (**M**) H_2_O_2_. (**II**) Statistical analysis was performed using one-way ANOVA followed by the Tukey P test with multiple comparisons: * *p* < 0.05 and ** *p* < 0.01.

Co-administration of FA–DOC–ERL NLCs showed increased green fluorescence, indicating increased cell death compared to pure drugs, unconjugated NLCs, and single administration of FA NLCs (*p* < 0.01) (Figure 15).

## 7. Discussion

The oral route is widely favored for the administration of chronic treatments for conditions such as cancer, heart disease, and diabetes due to its high patient acceptability. However, it poses challenges for the delivery of poorly water-soluble drugs, which spurred the development of lipid-based nanoparticles, significantly enhancing the oral absorption of poorly water-soluble drugs and leading to improved bioavailability [36]. In this study, we prepared FA–DOC–ERL NLCs and investigated their therapeutic efficacy compared to pure drugs.

The solid and liquid lipids play a crucial role in the preparation of NLCs as they encapsulate drugs and prevent their partitioning from the lipidic matrix into the surrounding aqueous environment [16]. The incorporation of a liquid lipid was observed to form more imperfect lipid matrices, allowing greater accommodation of drugs. Mono- and di-glycerides within lipid matrices enhance the solubilization of poorly water-soluble drugs, given their emulsification properties [37,38]. Precirol ATO 5 and Labrafil M2125Cs have the highest percentage of monoglycerides, thereby displaying greater affinity for DOC and ERL [13]. However, the addition of liquid lipid beyond its solubility results in improper mixing and phase separation, forming nano-compartments of liquid lipids encircling the solid matrix [39]. DSC studies revealed that the 7:3 ratio lowered the melting point to 65.07 °C compared to other BM with respect to solid lipid (66.12 °C), confirming the proper incorporation of liquid lipid into the imperfect crystalline structure of the lipidic matrices [14]. Incorporating a larger amount of liquid lipid into the matrices enhances the NLCs’ loading capacity [37]. Surfactants also play a role in NLC development. They reduce lipid–aqueous interfacial tension and maintain colloidal repulsion between NLC particles [40]. Surfactants with HLB value > 10 significantly emulsify lipids into the aqueous media, providing stability to the NLCs. Tween 20 (HLB = 16.2), containing a tetrahydrofuran ring and polyoxyethylene chain, imparts steric stabilization to the NLCs. The dense hydrophobic tail keeps nanoparticles separate, avoiding aggregate formation and introducing electrostatic repulsion among the particles, further stabilizing the NLCs. Additionally, being a non-ionic surfactant, the negative charge on the NLCs is attributed to the ionization of the long alkyl chain fatty acids from the glycerides present in the lipids [41,42]. A drug–excipient compatibility study ensured the selection of suitable excipients for the development of stable NLCs. The FTIR analysis indicated no significant changes in the drug peaks, suggesting the absence of chemical incompatibility between the drugs and the chosen excipients. However, minor changes were observed within the spectra of the physical mixtures, such as the peaks’ broadening and slight shifting of the peaks due to the mixing and dilution effects.

The QbD approach was employed for the optimization of NLCs. The parameters significantly impacting the process performance were identified by PBD, and their interactions were analyzed using BBD [43]. It was observed that during sonication, 40% amplitude was insufficient in reducing the size, and increasing the amplitude to 80% resulted in reduced stability due to enhancement in cavitation energy, leading to agglomeration. Hence, 60% amplitude was optimized for NLCs. The actual vs. predicted plot demonstrated that the designed model had a significant (*p* < 0.05) effect over all the responses. Three factors, namely, concentration of surfactant, BM, and sonication time, exhibited a significant effect (*p* < 0.05) on defined responses. From the Pareto chart of the BBD, surfactant concentration was considered a major contributing factor in evaluating all the responses. The prediction profiler and response plots revealed that the size increases with increased BM concentration. At increased concentration, the lipids agglomerate and increase the viscosity of the lipid phase, resulting in rapid solidification of the lipids, which might influence the probe sonicator’s shearing ability. This also results in an increased PDI. EE and DL were also increased with increasing BM concentration, due to the availability of more monoglycerides of lipids within the spare space of the matrices, facilitating enhanced encapsulation. The augmentation of surfactant concentration resulted in the reduction of NLC size. The elevated surfactant concentration provides a greater number of surfactant molecules, forming a protective layer over the expanded surface area of nanoparticles. This shielding effect impedes the amalgamation of particles into larger entities. Additionally, the increased presence of surfactants on the surface diminishes the interfacial tension between the lipid and aqueous phases, playing a role in facilitating the formation of solid particles of the NLCs.

Elevating surfactant concentration enhances drug partitioning in the surfactant layer, limiting drug encapsulation and loading within the lipid matrices. Sonication time negatively influences PS and PDI due to heightened mechanical energy preventing particle agglomeration. However, excessive energy input can lead to instability and coalescence of smaller particles into larger ones. Despite its impact on size, sonication time insignificantly affects EE and DL. The negative zeta potential of NLCs is attributed to Tween 20 surrounding the lipidic matrices, impeding particle mobility. The negative charge is linked to preferential -OH adsorption from water molecules onto the lipid particles. Similar results were reported by Sabzichi et al. 2017 [44]. A marginal increase in PS and a decrease in ZP serve as confirmation of FA attachment to the surface of the NLCs. This attachment was further validated through FTIR analysis. The presence of FA also contributes to the broadening of the PDI, likely indicative of aggregated formations with surrounding particles. These findings align with the results reported by Oshiro-Junior et al. [45]. Freeze-drying enhances the stability of NLCs by establishing a protective network around NLCs. However, this process can lead to an increase in size due to nanoparticle aggregation caused by alterations in the surfactant’s properties resulting from the removal of water molecules. To mitigate such changes, the addition of a suitable cryoprotectant is essential. Fructose at 5 *w*/*v* serves this purpose, and observation aligns with similar findings reported in our previous studies [46,47].

To analyze the drug release profile from NLCs, the developed NLCs were exposed to different release media mimicking various physiological conditions and a tumor microenvironment. As anticipated, ~10% release occurred in pH 1.2 for all formulations. However, the initial release observed might be attributed to the presence of unentrapped drugs on the NLCs’ surface. In pH 6.8 and 7.4, the NLCs exhibited a biphasic release profile, characterized by an initial burst release followed by sustained release. This sustained release was attributed to the release of the drugs entrapped within the lipid matrices, facilitated by the erosion of the lipidic core, allowing diffusion of the aqueous media and enhancing drug dissolution. These findings align with a study by Saif et al. in 2020 [48]. In a tumor microenvironment (pH 5.5), the drugs, being basic, undergo protonation (NH to -NH_2_^+^), which strengthens the hydrogen bonding, facilitating the translocation of the drugs into the aqueous channels of the NLCs. This increases the hydrophilicity and solubility of the drugs, leading to their rapid release. Conversely, at pH 7.4, the protonated fraction of the drugs decreases while the zwitterionic fraction increases, rendering the drugs more lipophilic, resulting in delayed drug release compared to pH 5.5. Similar release profiles were observed by Teixeira et al. [49]. The alteration in the surface characteristics of the NLCs by FA conjugation decreased the release of the drugs due to the barrier provided by the FA and the associated ligand corona on the nanoparticles [50]. Slightly higher release of drugs was observed from FA NLCs at pH 5.5 compared to NLCs due to the partial deprotonation of the -COOH group of FA in pH 5.5 [51].

The release kinetics of drugs from NLCs were well explained by their composition and the structure of the particle. The kinetic models inferred that NLCs follow a Higuchi model, indicating drug diffusion from the matrix system, consistent with the findings of Elmowafy et al. [52]. FA NLCs follow the Korsmeyer–Peppas model, which indicates surface erosion-based drug release from the system, in accordance with the findings of Sahrayi et al. [53]. From the Korsmeyer–Peppas model, it was observed that the release mechanism in NLCs was governed by diffusion, while FA NLCs follow non-Fickian transport, which means the release process is a combination of diffusion and erosion.

pH-stat lipolysis was utilized to investigate the fate of NLCs during digestion in the GIT. The digestion of lipid-based formulations releases fatty acids, leading to a decrease in pH. Bile salts and phospholipids in the GIT convert free fatty acids into micelles, theoretically serving as carriers for lipids and therapeutic agents. These micelles are then transported collectively to epithelial cells for absorption. The presence of Labrafil M2125Cs (a mixture of medium-chain triglycerides) leverages their fast digestion, enhancing drug bioaccessibility by migrating to the surrounding aqueous phase. The FA coating slows down lipolysis due to the physical barrier formed by the FA shell around the NLCs. The intactness of the NLCs increases their chances of absorption via endocytosis or transcytosis. The bioaccessibility of DOC and ERL significantly improved with emulsification compared to their suspensions, attributed to the increased surface area-to-volume ratio enhancing lipid digestion and drug diffusion into micelles. However, fewer drugs were found to be bioaccessible from FA NLCs due to decreased lipolysis, resulting in reduced drug availability in the aqueous phase and more intact NLCs for lymphatics. These findings align with a study conducted by Aditya et al. [54].

The NLCs were stable at 4 °C and 25 ± 2 °C/60 ± 5%RH for at least six months, consistent with the finding of Thiruchenthooran et al. [55]. For the stability profile of NLCs in different buffers, a common outcome is dissolution followed by possible re-precipitation and aggregation. Therefore, the particle size might initially decrease and then potentially increase. A similar result was obtained by Wu et al. [26].

The cytotoxicity profile of drugs and NLCs was assessed in MDA-MB-231 and 4T1 cell lines using MTT assays. The treatment with NLCs exhibited time- and concentration-dependent cytotoxicity. The IC_50_ values were found to be in the order of FA NLCs < NLCs < free drugs, demonstrating the influence of FA exposure on the surface of NLCs. The combination of drugs at a molar ratio of 1:3 (DOC:ERL) demonstrated the most robust synergism. The FA–DOC–ERL NLCs selectively bind to the overexpressed folate receptors, facilitating increased internalization through caveolar-based receptor-mediated endocytosis. These results align with a previous study by Kim et al. [56], and Varshosaz et al. [57]. Disruption of the mitochondrial membrane leads to a loss of mitochondrial potential, causing less accumulation of JC-1 dyes within the mitochondria, as evidenced by the shift of red to green fluorescence. Cells with FA–DOC–ERL NLCs displayed increased green fluorescence, suggesting that FA NLCs disrupted the mitochondrial membrane, resulting in increased apoptosis. The results were in concordance with the findings of Pal et al. [58]. The increased internalization of DOC and ERL through the FA receptors on the surface of the cells decreased their colony-forming ability compared to free drugs. Such results demonstrate the efficacy of the co-administration of FA–DOC–ERL NLCs in restraining the metastatic possibility associated with improved intracellular accumulation of drugs with nanoformulations [59]. MDA-MB-231 breast cancer cells are highly metastatic due to the presence of metastatic proteins like MRTF-a and STAT3. The co-administration of FA–DOC–ERL NLCs showed increased cellular uptake, inhibiting cell migration down the well [60]. H_2_DCFDA dye (non-fluorescent) converts into DCF (green fluorescent) upon ROS generation. The higher green fluorescence upon co-administration of FA–DOC–ERL NLCs demonstrates the higher uptake of drugs, facilitating enhanced cytotoxicity. This observation indicates a correlation of the ROS-mediated cytotoxicity in MDA-MB-231 cell lines [61]. The in vitro results were in accordance with our previous report [18].

## 8. Conclusions

In this study, folic acid-conjugated DOC–ERL NLCs were successfully prepared using the hot melt homogenization–ultrasonic dispersion method, and their formulation was optimized through Plackett–Burman design (PBD) and Box–Behnken design (BBD). The resulting nanoparticles exhibited desirable characteristics: spherical, small (<200 nm), PDI < 0.30, ZP < −30 mV, high EE (~95%), and moderate drug loading (~5%). Both NLCs and FA NLCs demonstrated excellent storage stability at 4 °C and 25 ± 2 °C/60 ± 5%RH for six months. The in vitro drug release study revealed a sustained release profile of drugs from NLCs over 24 h, while the in vitro digestion study indicated the bioaccessibility of DOC and ERL in intestinal media after encapsulation in NLCs. The co-administration system of DOC and ERL using FA NLCs was successfully established with an optimized synergistic molar ratio of 1:3 (DOC:ERL). In TNBC cells, the combination of DOC and ERL delivered through FA NLCs exhibited significant synergistic anticancer activity, as demonstrated by MTT, MMP, and ROS assays. Furthermore, the combination also hindered the migratory characteristics of TNBC, evident from assays such as colony formation and transwell migration. Thus, the co-administration of DOC and ERL using FA NLCs represents a promising approach for TNBC treatment. However, further in vivo studies are warranted to validate the efficacy of the developed system in a real biological environment.

## Figures and Tables

**Figure 3:**
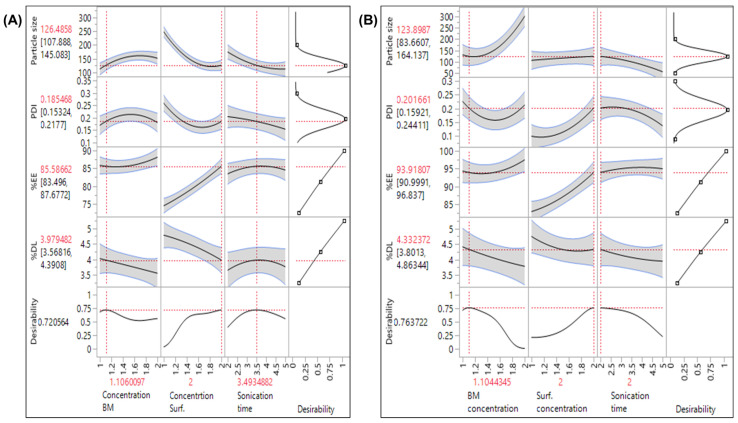
Prediction profiler depicting the maximum desirability at 95% confidence level for DOC NLCs (**A**) and ERL NLCs (**B**), revealing the influence of selected factors on responses. The gray color depicts the region ranging between the low and high values of the responses, marked by the blue line. The black line marks the optimum data. The dashed red line extrapolated the optimum response with x axis and y axis.

**Table 1:** Variables with the assigned values at different levels in the PDB and BBD.

Plackett–Burman Design (PBD)				
Factors	Levels			
	Level 1		Level 2	
BM (%*w*/*v*)	1		2	
Surfactant (%*w*/*v*)	1		2	
Mixing speed (rpm)	250		750	
Homogenization time (min)	1		5	
Homogenization speed (rpm)	1000		5000	
Sonication time (min)	2		5	
Sonication amplitude (%)	40		80	
Responses	Constraints			
Size (nm)	<200			
PDI	<0.35			
Box–Behnken Design (BBD)				
Factors	Levels			
	Low (−1)	Intermediate (0)		High (+1)
BM (%*w*/*v*)	1	1.5		2
Surfactant (%*w*/*v*)	1	1.5		2
Sonication time (min)	2	3.5		5
Responses	Constraints			
Size (nm)	<200			
PDI	<0.35			
Entrapment efficiency (%EE)	Maximum			
Drug loading (%DL)	Maximum			

**Table 2:** Detailed experimental batches of DOC NLCs in BBD.

S. No.	Conc. BM (%*w*/*v*)	Conc. Surf (%*w*/*v*)	Sonication Time (min)	Particle Size (nm)	PDI	EE (%)	DL (%)
1	2	1.5	2	189.42 ± 2.42	0.278 ± 0.003	84.12 ± 1.24	3.62 ± 0.02
2	2	2	3.5	158.11 ± 1.32	0.167 ± 0.001	87.76 ± 2.11	3.39 ± 0.04
3	1.5	1.5	3.5	156.46 ± 1.36	0.211 ± 0.002	81.15 ± 1.67	4.14 ± 0.02
4	2	1	3.5	245.62 ± 2.77	0.344 ± 0.003	85.54 ± 1.48	4.08 ± 0.03
5	1.5	1	2	300.34 ± 3.17	0.329 ± 0.002	76.75 ± 1.35	4.54 ± 0.07
6	2	1.5	5	141.67 ± 4.16	0.156 ± 0.006	84.42 ± 1.27	3.62 ± 0.05
7	1	1.5	5	132.81 ± 2.49	0.125 ± 0.005	78.63 ± 2.03	3.89 ± 0.08
8	1.5	1.5	3.5	178.42 ± 2.16	0.232 ± 00.004	80.34 ± 2.16	4.12 ± 0.04
9	1.5	2	2	200.36 ± 2.37	0.254 ± 0.001	84.84 ± 1.86	3.64 ± 0.01
10	1	1.5	2	180.21 ± 1.65	0.134 ± 0.002	76.31 ± 1.53	3.34 ± 0.05
**11**	**1**	**2**	**3.5**	**123.80 ± 2.10**	**0.175 ± 0.004**	**78.68 ± 1.41**	**4.09 ± 0.01**
12	1	1	3.5	231.57 ± 3.08	0.245 ± 0.007	85.54 ± 1.37	3.12 ± 0.03
13	1.5	1.5	3.5	164.32 ± 2.64	0.209 ± 0.008	82.34 ± 1.26	3.41 ± 0.01
14	1.5	1	5	268.16 ± 2.81	0.314 ± 0.003	75.53 ± 2.33	3.54 ± 0.02
15	1.5	2	5	146.23 ± 2.58	0.166 ± 0.002	84.03 ± 1.42	3.67 ± 0.06

EE = entrapment efficiency; DL = drug loading. Values are presented as means ± SD (n = 3). The bold format suggests the optimized formulation.

**Table 3:** Detailed experimental batches of ERL NLCs in BBD.

S. No.	Conc. BM (%*w*/*v*)	Conc. Surf (%*w*/*v*)	Sonication Time (min)	Particle Size (nm)	PDI	EE (%)	DL (%)
1	2	1.5	5	210.23 ± 1.65	0.208 ± 0.002	94.84 ± 1.33	3.64 ± 0.02
2	2	1	3.5	289.42 ± 2.43	0.291 ± 0.004	95.39 ± 1.14	4.08 ± 0.05
3	1.5	1.5	3.5	132.11 ± 2.74	0.113 ± 0.002	90.94 ± 1.25	3.74 ± 0.04
4	1.5	1.5	3.5	135.27 ± 2.12	0.122 ± 0.005	89.20 ± 1.38	4.03 ± 0.03
5	2	2	3.5	237.82 ± 1.44	0.225 ± 0.003	95.76 ± 1.05	3.39 ± 0.01
6	1	1.5	2	130.35 ± 1.31	0.128 ± 0.006	86.30 ± 2.41	3.34 ± 0.04
7	2	1.5	2	311.19 ± 1.57	0.200 ± 0.001	94.10 ± 2.15	3.62 ± 0.02
8	1	1	3.5	150.74 ± 2.17	0.146 ± 0.002	84.13 ± 1.22	4.04 ± 0.06
**9**	**1.5**	**2**	**2**	**120.57 ± 2.76**	**0.213 ± 0.003**	**95.53 ± 1.52**	**4.13 ± 0.03**
10	1.5	1	5	145.64 ± 2.83	0.178 ± 0.005	85.76 ± 1.73	3.64 ± 0.07
11	1.5	2	5	168.37 ± 1.58	0.196 ± 0.002	94.03 ± 1.74	3.47 ± 0.03
12	1	1.5	5	138.60 ± 1.22	0.109 ± 0.004	88.68 ± 1.29	3.43 ± 0.05
13	1.5	1	2	221.24 ± 1.40	0.225 ± 0.003	86.75 ± 1.14	4.35 ± 0.06
14	1.5	1.5	3.5	135.29 ± 2.47	0.132 ± 0.001	89.20 ± 1.20	4.01 ± 0.02
15	1	2	3.5	128.51 ± 2.50	0.233 ± 0.002	94.51 ± 2.11	4.09 ± 0.01

EE = entrapment efficiency; DL = drug loading. Values are presented as means ± SD (n = 3). The bold format suggests the optimized formulation. Maximum desirability was obtained by plotting the prediction profiler (Figure 3A,B). The concentration of BM and surfactant showed a parabolic effect on all the responses and significantly affected the responses at lower and higher values. On the contrary, sonication time showed a parabolic effect on particle size and PDI, but an insignificant effect on EE and DL. The concentration of BM and surfactant significantly affected all the responses for DOC–ERL NLCs, whereas sonication times primarily affected the particle size and PDI for DOC–ERL NLCs. The maximization of desirability for DOC NLCs was represented by 1% *w*/*v* BM, 2% *w*/*v* surfactant, and 3.5 min sonication time, and for ERL NLCs, 1.5% *w*/*v* BM, 2% *w*/*v* surfactant, and 2 min sonication time at 95% confidence level.

**Table 4:** Model validation of Box–Behnken design.

Formulation	BM. Conc. (%*w*/*v*)	Surf. Conc. (%*w*/*v*)	Sonication Time (min)	Actual vs. Predicted	PS (nm)	PDI	%EE.	%DL
DOC NLCs	1	2	3.5	Predicted	126.48	0.185	85.58	3.97
				Actual	123.80	0.175	78.68	4.09
				Prediction error	2.12	5.41	8.06	2.93
ERL NLCs	1.5	2	2	Predicted	123.89	0.201	93.92	4.33
				Actual	120.57	0.213	95.53	4.13
				Prediction error	2.67	5.63	1.68	4.68

**Table 5:** Quality attributes of optimized formulations.

Parameters	PS (nm)	PDI	ZP (mV)	EE (%)	DL (%)	Height (nm) in Z Axis
DOC NLCs	123.80 ± 2.10	0.175 ± 0.004	−15.25 ± 0.69	78.68 ± 1.41	4.09 ± 0.01	56.29 ± 1.32
FA–DOC NLCs	153.2 ± 2.52	0.241 ± 0.005	−14.15 ± 0.45	80.56 ± 1.85	4.10 ± 0.05	38.76 ± 1.27
ERL NLCs	120.57 ± 2.76	0.213 ± 0.003	−16.44 ± 0.63	95.53 ± 1.52	4.13 ± 0.03	87.18 ± 1.42
FA–ERL NLCs	147.40 ± 3.32	0.258 ± 0.001	−15.86 ± 0.55	96.34 ± 1.55	4.20 ± 0.02	55.35 ± 1.38

Values are presented as means ± SD (n = 3).

**Table 6:** IC_50_ values of the combinations in MDA-MB-231 and 4T1 cells.

Combinations	IC_50_ Values (μM) at 24 h	
	MDA-MB-231	4T1
DOC + ERL	7.51 ± 0.87	8.42 ± 0.65
DOC NLCs + ERL NLCs	1.54 ± 0.27	1.79 ± 0.38
FA–DOC NLCs + FA–ERL NLCs	0.71 ± 0.06	0.81 ± 0.03

## Data Availability

The authors confirm that the data for this study are available within the article and the Supplementary Materials.

## Supplementary Materials

The following supporting information can be downloaded at: https://www.mdpi.com/article/10.3390/pharmaceutics16070926/s1, Figure S1: Ishikawa fishbone diagram displaying the interaction between CMA (critical material attribute) and CPP (critical process parameters) to optimize CQA (critical quality attributes); Figure S2: (A) Schematic Diagram of synthesis of Folic acid–Stearic acid (FA-SA) conjugate. (B) FTIR spectra of Folic acid (FA), stearic acid (SA), physical mixture of FA, and SA (FA + SA), and conjugated FA, and SA (FA-SA); Figure S3: DSC thermograms of BM and Precirol ATO 5; Figure S4: FTIR spectra indicating the compatibility profile of various excipients of NLCs with DOC (A) and ERL (B); Figure S5: 3D response surface plot representing the influence of factors on PS (A–C), PDI (D–F), %EE (G–I), and %DL (J–L) for DOC-NLCs; Figure S6: 3D response surface plot representing the influence of factors on PS (A–C), PDI (D–F), %EE (G–I), and %DL (J–L) for ERL-NLCs; Figure S7: FTIR characterization of folic acid on the surface of DOC-NLCs (A), and ERL-NLCs (B); Figure S8: The particle size changes of NLCs in pH 1.2 (A), pH 5.5 (B), pH 6.8 (C), and pH 7.4 (D) for one week at 4 °C, respectively (mean ± SD, n = 3); Figure S9: Cell cytotoxicity assay in the MDA-MB-231 cell line for DOC, DOC-NLCs, and FA-DOC-NLCs at 24 (A), 48 (B), and 72 h (C), and ERL, ERL-NLCs, and FA-ERL-NLCs at 24 (D), 48 (E), and 72 h (F); Figure S10: Cell cytotoxicity assay in the 4T1 cell line for DOC, DOC-NLCs, and FA-DOC-NLCs at 24 (A), 48 (B), and 72 h (C), and ERL, ERL-NLCs, and FA-ERL-NLCs at 24 (D), 48 (E), and 72 h (F); Table S1: Detailed experimental batches of NLCs in PBD; Table S2: Preliminary Screening of different cryoprotectants based on re-dispersity index, and re-constitutional score for lyophilization of different NLCs formulations; Table S3: Optimization of Fructose concentration based on Re-dispersibility Index and Reconstitution Score for lyophilization of different NLCs formulations; Table S4: Kinetic Models for the analysis of release profile; Table S5: Stability of different NLCs formulations upon storage at different storage conditions of 4 °C, 25 ± 2 °C/60 ± 5%RH, and 40 ± 2 °C/75 ± 5%RH up to six months, as determined by the change of critical quality attributes.
