# Supplementary material for: Combinatorial Delivery of Docetaxel- and Erlotinib-Loaded Functionalized Nanostructured Lipid Carriers for the Treatment of Triple-Negative Breast Cancer Using Quality-by-Design Approach
Source: Pharmaceutics. 2024 Jul 11;16(7):926. doi: 10.3390/pharmaceutics16070926 (PMC11279545; doi:10.3390/pharmaceutics16070926)
Supplement: Supplementary file 1 [file pharmaceutics-16-00926-s001.zip › pharmaceutics-3074136-supplementary.pdf]

Supplementary Sheet

Tables

Table S1. Detailed experimental batches of NLCs in PBD.

S.No.	Conc. BM (%w/v)	Conc. Surf (%w/v)	Mixing speed (rpm)	Homo. Time (min)	Homo. Speed (rpm)	Sonication Time (min)	Sonication amplitude (%)	Particle Size (nm)	PDI
1	1.5	1	500	1	5000	3.5	80	255	0.275
2	2	2	500	5	1000	2	80	175	0.208
3	2	1.5	250	3	5000	3.5	60	232	0.212
4	1.5	1.5	250	1	1000	2	40	247	0.277
5	2	1	750	1	3000	5	40	223	0.223
6	1	2	250	5	5000	5	40	272	0.172
7	1	1	500	3	5000	2	40	302	0.312
8	2	1	250	3	1000	5	80	224	0.164
9	2	1.5	500	5	3000	3.5	40	209	0.205
10	1	2	250	1	3000	3.5	80	123	0.123
11	1	1.5	750	3	3000	2	80	174	0.164
12	1.5	1	250	5	3000	2	60	251	0.321
13	2	2	750	1	5000	2	60	198	0.198
14	1.5	2	500	3	3000	5	60	210	0.127
15	1.5	1.5	750	5	5000	5	80	167	0.167
16	1	1.5	500	1	1000	5	60	85	0.108
17	1	1	750	5	1000	3.5	60	113	0.217
18	1.5	2	750	3	1000	3.5	40	205	0.132

Conc. BM = Concentration of Binary Mixture; Conc. Surf = Concentration of surfactant; Homo. = Homogenization; PDI = Polydispersity Index

Table S2. Preliminary Screening of different cryoprotectants based on re-dispersity index, and re-constitutional score for lyophilization of different NLCs formulations.

	**DOC-NLCs**			**FA-DOC-NLCs**			**ERL-NLCs**			**FA-ERL-NLCs**		
	PS (nm)	Ri = (Sf/Si)	RS	PS (nm)	Ri = (Sf/Si)	RS	PS (nm)	Ri = (Sf/Si)	RS	PS (nm)	Ri = (Sf/Si)	RS
Ini. Size	123.80 ±2.10	-	-	153.20 ±2.90	-	-	120.57 ±2.40	-	-	147.40 ±2.30	-	-
D	324.23 ±3.07	2.63 ±0.11	**	435.76 ±2.40	2.84 ±0.17	**	367.23 ±2.34	3.04 ±1.14	**	378.44 ±2.52	2.56 ±0.16	**
S	211.06 ±2.15	1.71 ±0.21	**	411.23 ±2.65	2.68 ±0.05	**	312.78 ±3.20	2.59 ±1.12	**	435.23 ±2.16	2.95 ±0.05	**
T	231.54 ±3.17	1.87 ±0.13	**	325.21 ±2.32	2.12 ±0.16	**	222.43 ±3.10	1.84 ±1.03	**	318.33 ±3.32	2.15 ±0.17	**
M	354.33 ±4.08	2.87 ±0.17	**	232.54 ±3.43	1.51 ±0.13	**	265.45 ±3.25	2.20 ±0.09	**	278.23 ±4.14	1.88 ±0.06	**
**F**	**154.45 ±3.12**	**1.25 ±0.10**	***	**178.23 ±2.43**	**1.16 ±0.12**	***	**142.54 ±2.28**	**1.18 ±0.08**	***	**167.34 ±2.19**	**1.13 ±0.09**	***

PS: particle size; Ri: re-dispersibility index; RS: reconstitution score; D: Dextrose; S: Sucrose; T: Trehalose; M: Mannitol; F: Fructose. *** re-dispersible within 20 s with mild mixing, ** re-dispersible within 1 min. Bold indicates the optimized one. Values are presented as mean ±SD (n = 3).

Table S3. Optimization of Fructose concentration based on Re-dispersibility Index and Reconstitution Score for lyophilization of different NLCs formulations.

	DOC-NLCs			FA-DOC-NLCs			ERL-NLCs			FA-ERL-NLCs		
	PS (nm)	Ri = (Sf/Si)	RS	PS (nm)	Ri = (Sf/Si)	RS	PS (nm)	Ri = (Sf/Si)	RS	PS (nm)	Ri = (Sf/Si)	RS
Ini. Size	123.80 ±2.10	-	-	153.20 ±2.90	-	-	120.57 ±2.40	-	-	147.40 ±2.30	-	-
2.5%	206.11 ±3.15	1.66 ±0.12	**	256.40 ±4.13	1.67 ±0.12	**	214.54 ±3.11	1.78 ±0.06	**	244.23 ±3.24	1.65 ±0.18	**
**5%**	**150.21 ±3.53**	**1.21 ±0.12**	***	**175.14 ±2.25**	**1.14 ±0.15**	***	**144.44 ±2.22**	**1.19 ±0.05**	***	**165.28 ±2.32**	**1.12 ±0.08**	***
7.5%	156.45 ±2.32	1.26 ±0.05	***	188.34 ±2.12	1.23 ±0.04	***	150.55 ±3.33	1.25 ±0.11	***	170.98 ±4.15	1.16 ±0.08	***
10%	162.22 ±3.27	1.31 ±0.11	***	189.45 ±2.42	1.24 ±0.03	***	158.67 ±2.51	1.32 ±0.09	***	177.23 ±3.52	1.20 ±0.19	***

PS: particle size; Ri: re-dispersibility index; RS: reconstitution score; *** re-dispersible within 20 s with mild mixing, ** re-dispersible within 1 min. Bold indicates the optimized one. Values are presented as mean ±SD (n = 3).

Table S4. Kinetic Models for the analysis of release profile.

**Models**	**Correlation Coefficient (R^2)**			
	DOC-NLCs	ERL-NLCs	FA-DOC-NLCs	FA-ERL-NLCs
Zero order	0.9115	0.8746	0.9009	0.8902
First order	0.9718	0.9464	0.9054	0.9089
Higuchi	**0.9763**	**0.9728**	0.9508	0.9442
Hixon – Crowell	0.9632	0.9478	0.9376	0.9327
Korsmeyer-Peppas (n)	0.9174 (0.3725)	0.9098 (0.3587)	**0.9515** (0.7625)	**0.9582** (0.7738)

Table S5. Stability of different NLCs formulations upon storage at different storage conditions of 4 °C, 25 ± 2 °C/60 ± 5% RH, and 40 ± 2 °C/75 ± 5% RH up to six months, as determined by the change of critical quality attributes.

Parameters	Months	DOC-NLCs	FA-DOC-NLCs	ERL-NLCs	FA-ERL-NLCs
**Temperature: 4°C**					
Particle size (nm)	0	148.30±2.22	170.62±3.76	140.52±2.54	161.35±2.33
	1	149.76±2.53	170.11±2.45	141.66±2.78	162.72±2.36
	3	149.15±2.27	171.43±2.32	142.47±2.22	162.17±2.24
	6	150.81±2.41	172.11±2.25	142.02±2.74	163.63±2.78
PDI	0	0.214±0.002	0.235±0.003	0.228±0.003	0.232±0.002
	1	0.214±0.003	0.236±0.004	0.228±0.005	0.233±0.005
	3	0.215±0.004	0.237±0.006	0.229±0.004	0.234±0.008
	6	0.217±0.001	0.238±0.008	0.230±0.007	0.235±0.002
%EE.	0	78.73±1.34	81.56±1.95	95.11±1.54	95.48±1.74
	1	78.42±1.22	81.26±1.46	94.76±1.29	95.22±1.77
	3	77.45±1.67	80.63±1.73	94.33±1.42	94.7±1.26
	6	77.13±1.73	80.04±1.85	93.71±1.84	94.13±1.29
ZP (mV)	0	-15.72±0.14	-14.25±0.18	-16.25±0.11	-15.26±0.16
	1	-15.43±0.24	-13.87±0.15	-16.11±0.25	-14.74±0.17
	3	-15.12±0.11	-13.45±0.21	-15.73±0.18	-14.15±0.21
	6	-14.78±0.23	-13.10±0.28	-15.54±0.17	-13.73±0.26
**Temperature: 25±2 ◦C; Relative humidity: 60±5% RH**					
Particle size (nm)	0	148.30±2.22	170.62±3.76	140.52±2.54	161.35±2.33
	1	150.70±4.45	172.71±2.35	142.66±4.78	163.62±4.56
	3	156.50±3.65	177.40±2.76	145.75±4.22	167.37±3.24
	6	161.80±3.33	182.30±4.29	150.62±3.74	172.43±3.78
PDI	0	0.214±0.002	0.235±0.003	0.228±0.003	0.232±0.002
	1	0.215±0.003	0.237±0.004	0.232±0.005	0.235±0.005

	3	0.218±0.002	0.239±0.006	0.233±0.004	0.236±0.008
	6	0.220±0.004	0.243±0.008	0.236±0.007	0.239±0.002
%EE.	0	78.73±1.34	81.56±1.95	95.11±1.54	95.48±1.74
	1	78.21±1.22	80.66±1.46	94.76±1.29	95.02±1.77
	3	77.45±1.67	80.23±1.73	94.13±1.42	94.12±1.26
	6	76.63±1.73	79.64±1.85	93.31±1.84	94.23±1.29
ZP (mV)	0	-15.72±0.14	-14.25±0.18	-16.25±0.11	-15.26±0.16
	1	-15.23±0.24	-13.72±0.15	-16.21±0.25	-14.81±0.17
	3	-14.32±0.11	-13.15±0.21	-16.23±0.18	-14.25±0.21
	6	-13.78±0.23	-12.12±0.28	-15.24±0.17	-13.53±0.26
**Temperature: 40±2◦C; Relative humidity: 75±5% RH**					
Particle size (nm)	0	148.30±2.22	170.62±3.76	140.52±2.54	161.35±2.33
	1	160.76±3.53	185.22±4.45	158.56±4.78	178.72±3.36
	3	178.15±4.27	206.45±4.32	178.44±3.22	202.43±3.24
	6	193.81±4.41	224.21±4.25	193.66±4.34	220.34±3.78
PDI	0	0.214±0.002	0.235±0.003	0.228±0.003	0.232±0.002
	1	0.235±0.003	0.257±0.004	0.252±0.005	0.265±0.005
	3	0.258±0.002	0.289±0.006	0.288±0.004	0.286±0.008
	6	0.274±0.004	0.303±0.008	0.292±0.007	0.310±0.002
%EE.	0	78.73±1.34	81.56±1.95	95.11±1.54	95.48±1.74
	1	76.73±1.22	79.66±1.46	93.31±1.29	91.88±1.77
	3	73.33±1.67	76.46±1.73	90.71±1.42	88.08±1.26
	6	69.13±1.73	73.36±1.85	87.21±1.84	84.28±1.29
ZP (mV)	0	-15.72±0.14	-14.25±0.18	-16.25±0.11	-15.26±0.16
	1	-13.43±0.24	-12.87±0.15	-15.11±0.25	-12.74±0.17
	3	-10.12±0.11	-10.45±0.21	-13.73±0.18	-9.15±0.21

	6	-8.78±0.23	-7.10±0.28	-10.54±0.17	-6.73±0.26

Figures

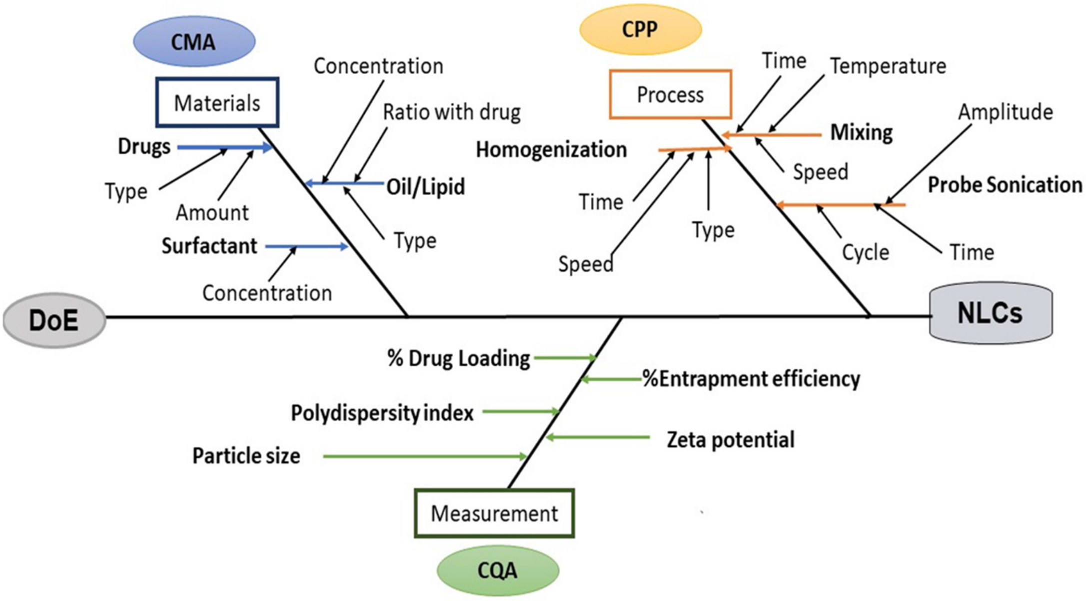

Figure S1. Ishikawa fishbone diagram displaying the interaction between CMA (critical material attribute) and CPP (critical process parameters) to optimize CQA (critical quality attributes). DoE: design of experiments.

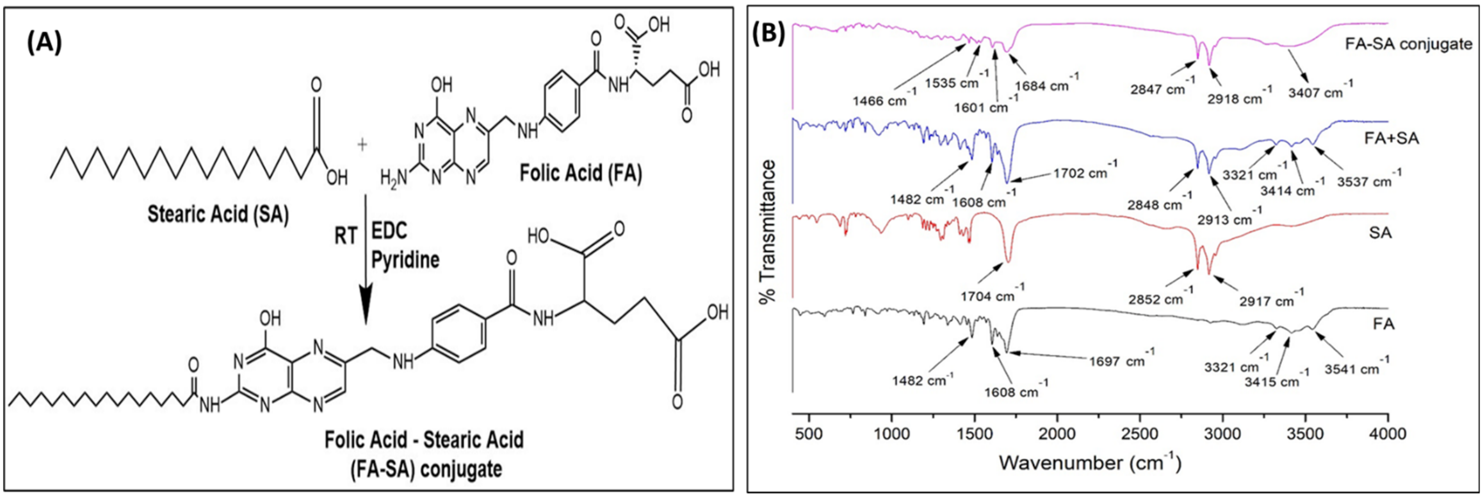

Figure S2. (A) Schematic Diagram of synthesis of Folic acid - Stearic acid (FA-SA) conjugate. (B) FTIR spectra of Folic acid (FA), stearic acid (SA), physical mixture of FA, and SA (FA + SA), and conjugated FA, and SA (FA-SA).

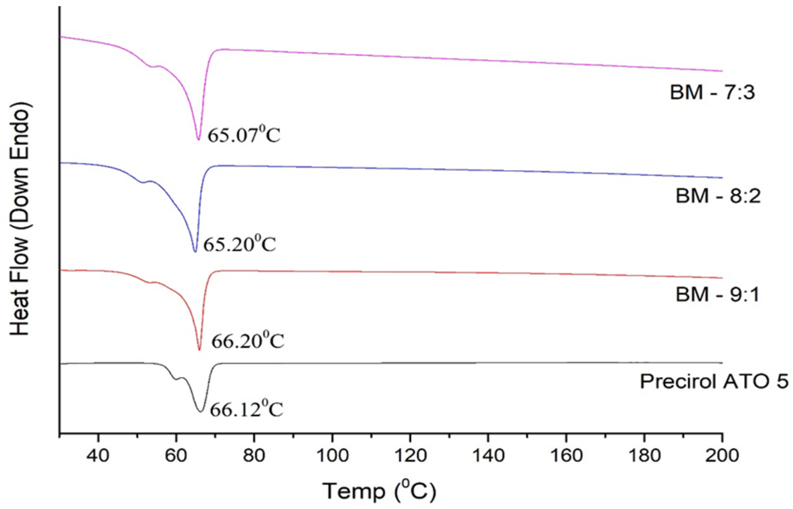

Figure S3. DSC thermograms of BM and Precirol ATO 5.

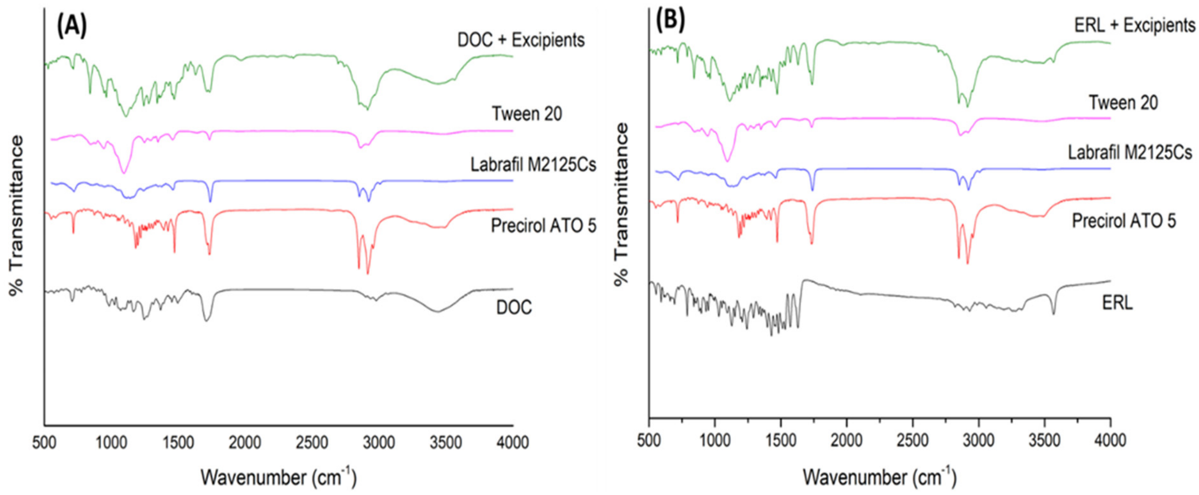

Figure S4. FTIR spectra indicating the compatibility profile of various excipients of NLCs with DOC (A) and ERL (B).

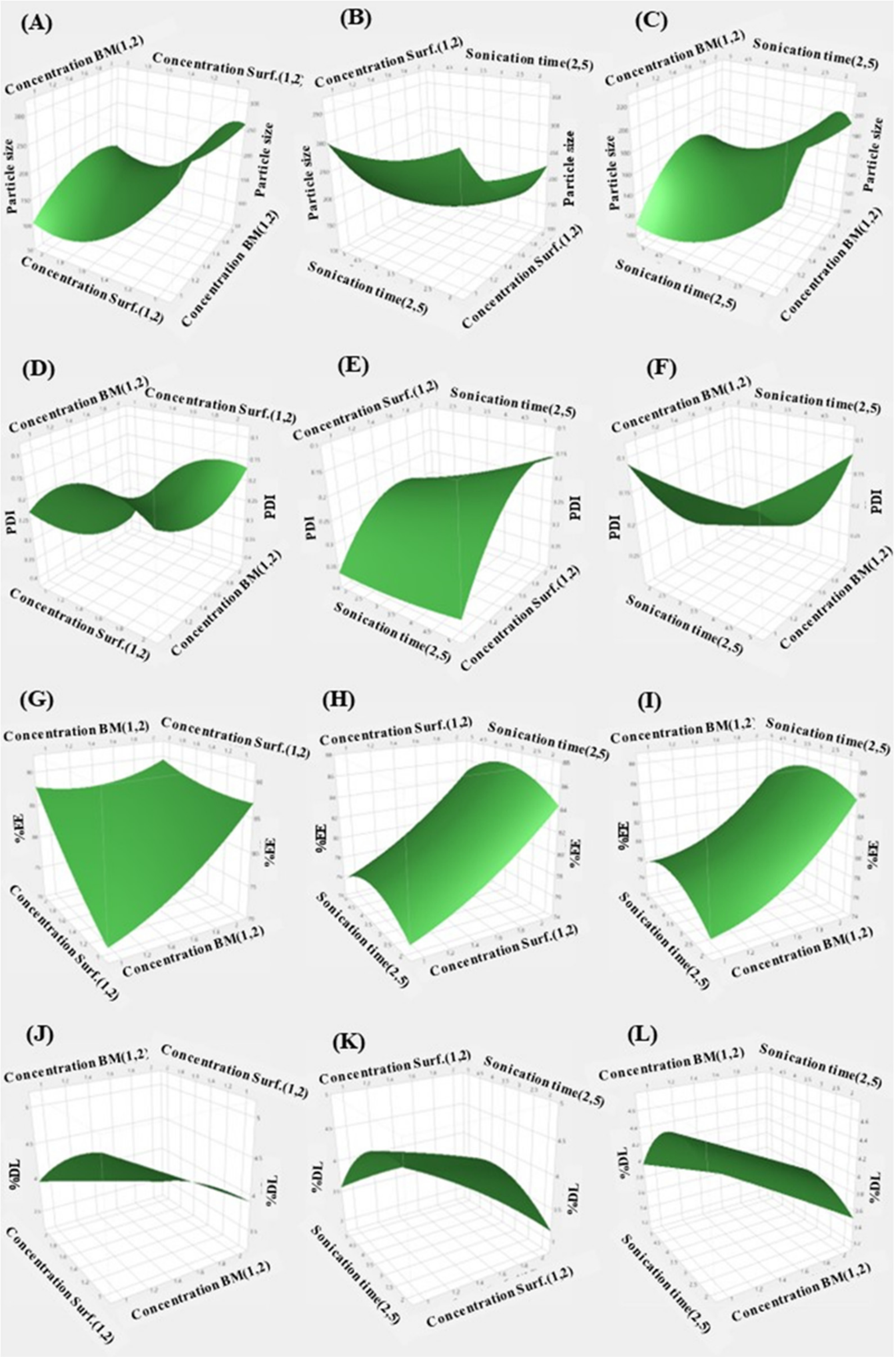

Figure S5. 3D response surface plot representing the influence of factors on PS (A–C), PDI (D–F), %EE (G–I), and %DL (J–L) for DOC-NLCs.

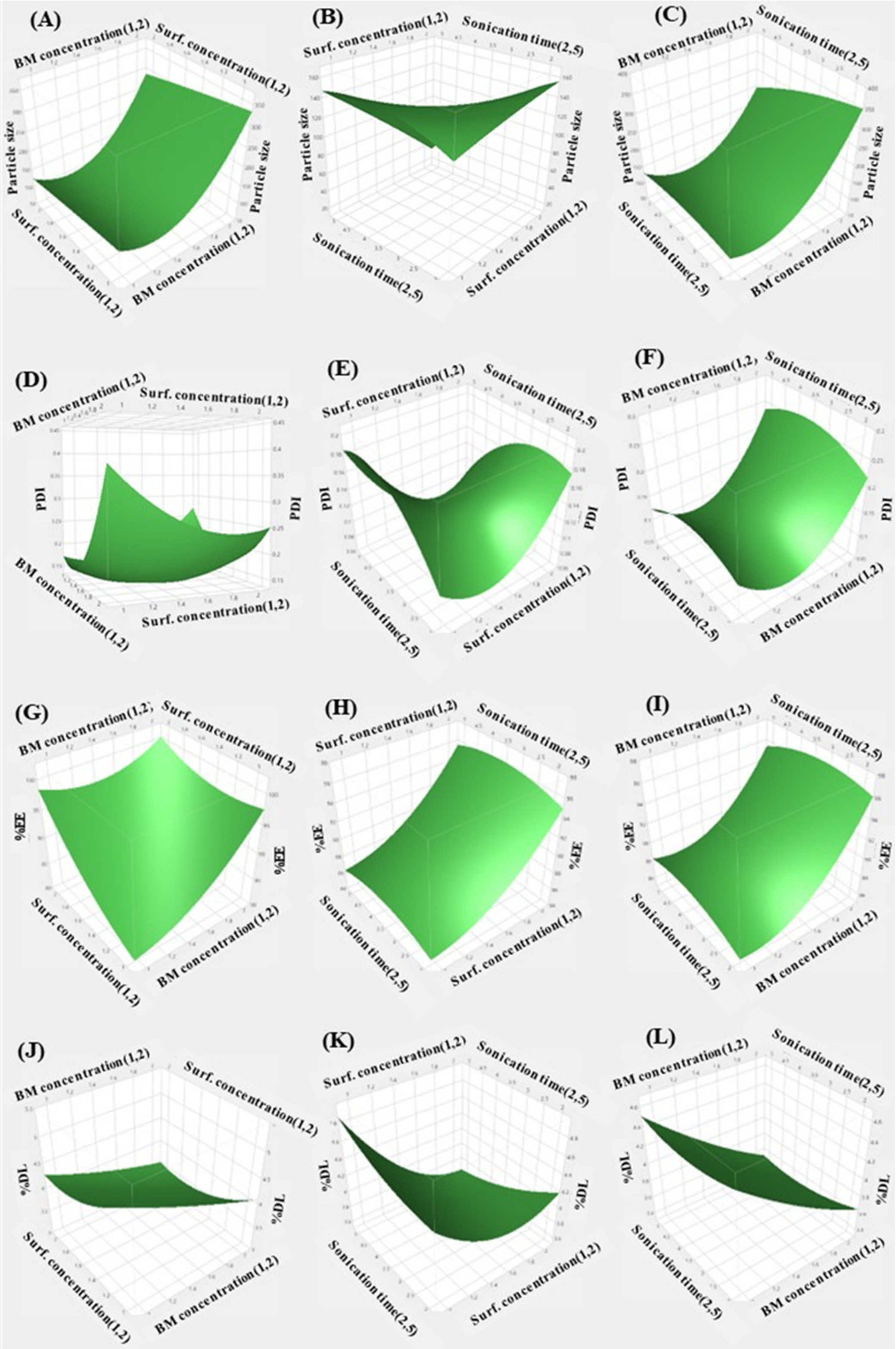

Figure S6. 3D response surface plot representing the influence of factors on PS (A–C), PDI (D–F), %EE (G–I), and %DL (J–L) for ERL-NLCs.

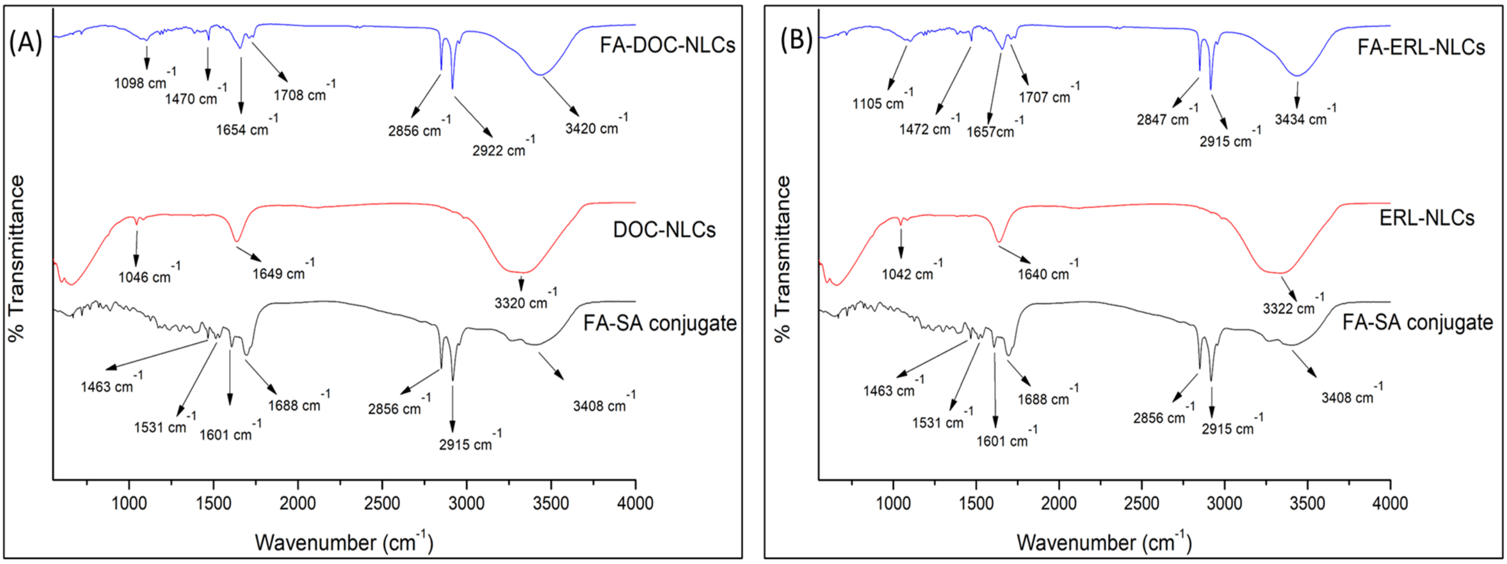

Figure S7. FTIR characterization of folic acid on the surface of DOC-NLCs (A), and ERL-NLCs (B).

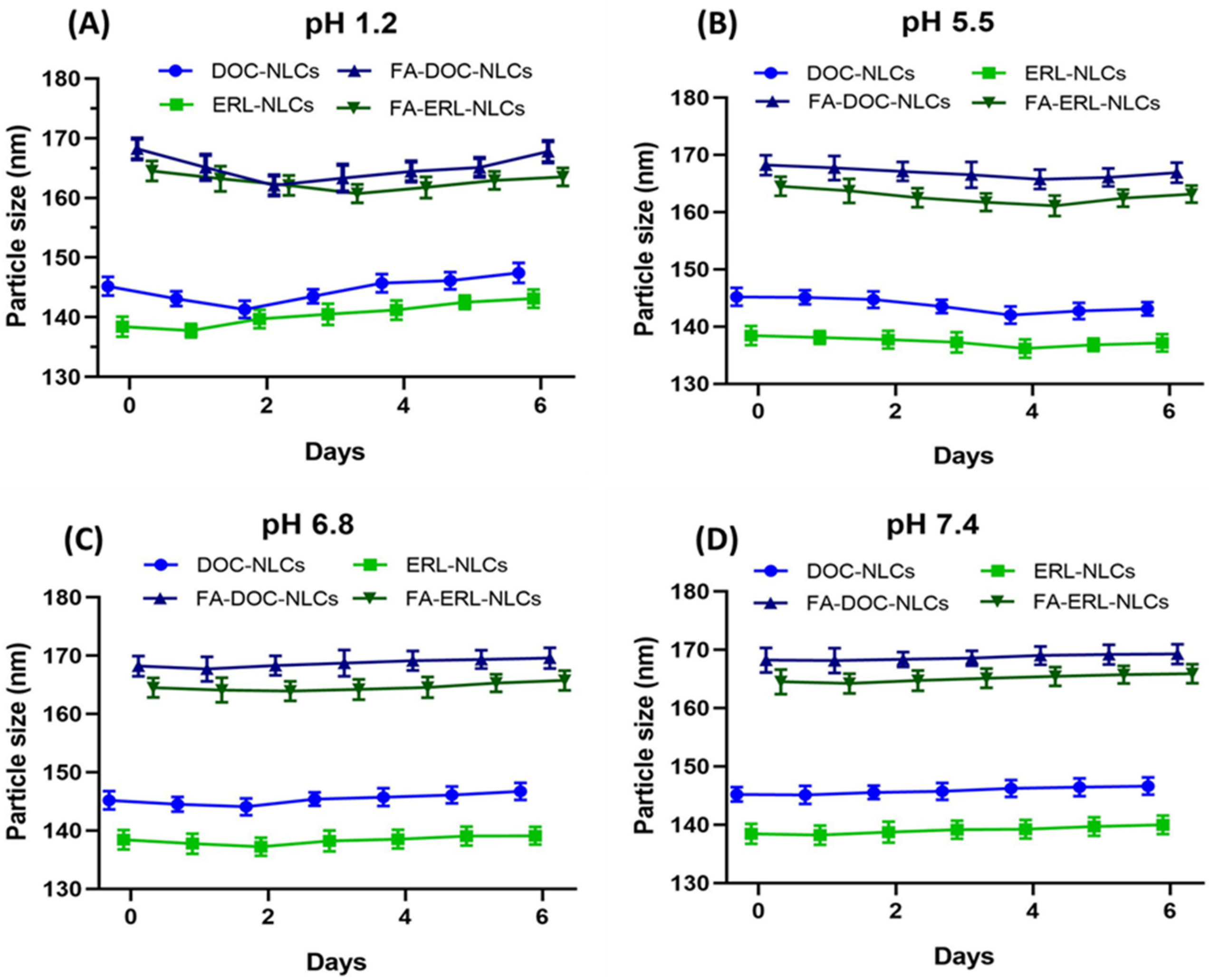

Figure S8. The particle size changes of NLCs in pH 1.2 (A), pH 5.5 (B), pH 6.8 (C), and pH 7.4 (D) for one week at 4 °C, respectively (mean ± SD, n = 3).

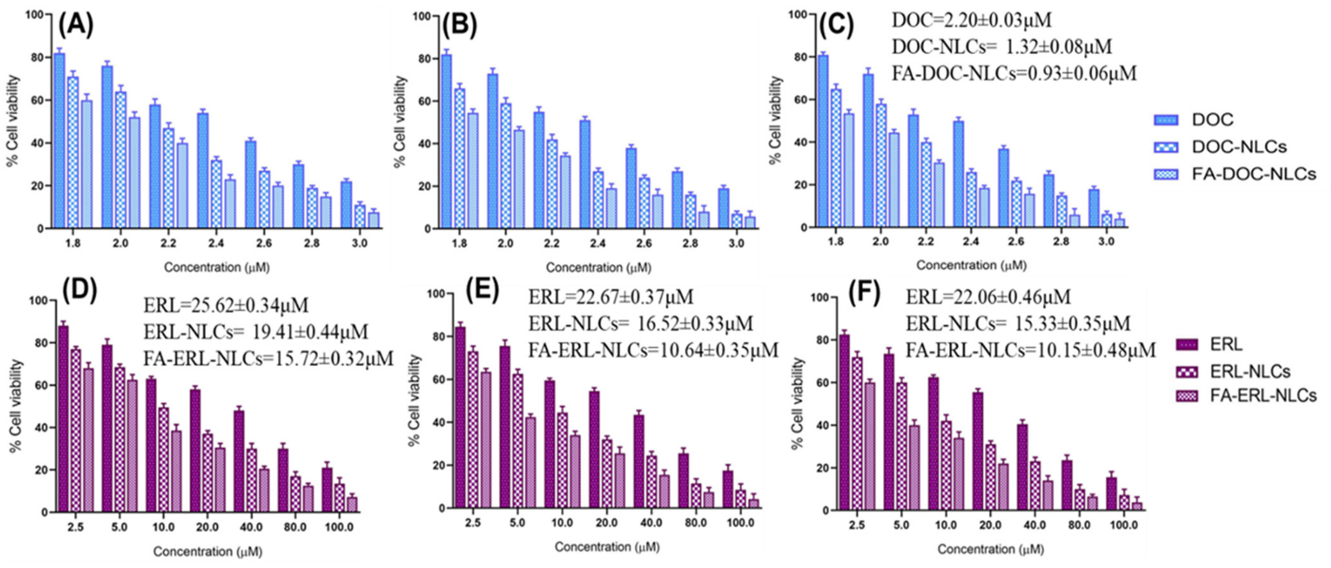

Figure S9. Cell cytotoxicity assay in the MDA-MB-231 cell line for DOC, DOC-NLCs, and FA-DOC-NLCs at 24 (A), 48 (B), and 72 h (C), and ERL, ERL-NLCs, and FA-ERL-NLCs at 24 (D), 48 (E), and 72 h (F).

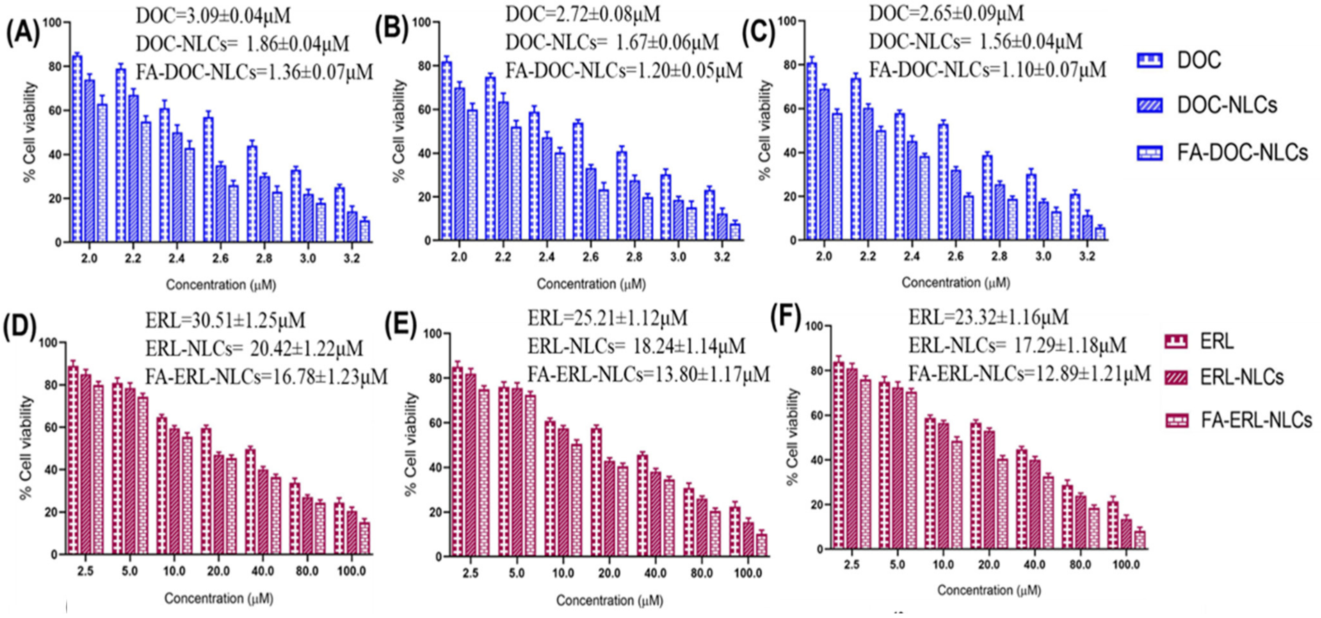

Figure S10. Cell cytotoxicity assay in the 4T1 cell line for DOC, DOC-NLCs, and FA-DOC-NLCs at 24 (A), 48 (B), and 72 h (C), and ERL, ERL-NLCs, and FA-ERL-NLCs at 24 (D), 48 (E), and 72 h (F).
